# Spatiotemporal spread of *Plasmodium falciparum* mutations for resistance to sulfadoxine-pyrimethamine across Africa, 1990–2020

**DOI:** 10.1371/journal.pcbi.1010317

**Published:** 2022-08-11

**Authors:** Jennifer A. Flegg, Georgina S. Humphreys, Brenda Montanez, Taryn Strickland, Zaira J. Jacome-Meza, Karen I. Barnes, Jaishree Raman, Philippe J. Guerin, Carol Hopkins Sibley, Sabina Dahlström Otienoburu

**Affiliations:** 1 School of Mathematics and Statistics, University of Melbourne, Melbourne, Victoria, Australia; 2 WorldWide Antimalarial Resistance Network (WWARN), Oxford, United Kingdom; 3 Green Templeton College, University of Oxford, Oxford, United Kingdom; 4 College of Science, Technology, Engineering and Mathematics, Johnson C. Smith University, Charlotte, *North Carolina*, United States of America; 5 Division of Clinical Pharmacology, Department of Medicine, University of Cape Town, Cape Town, South Africa; 6 WorldWide Antimalarial Resistance Network (WWARN) Pharmacology Scientific Working Group / Southern African Regional Centre, University of Cape Town, Cape Town, South Africa; 7 Centre for Emerging Parasitic and Zoonotic Diseases, National Institute for Communicable Diseases, Johannesburg, South Africa; 8 Wits Research Institute for Malaria, University of Witwatersrand, Johannesburg, South Africa; 9 Infectious Diseases Data Observatory (IDDO), Oxford, United Kingdom; 10 Centre for Tropical Medicine and Global Health, Nuffield Department of Clinical Medicine, University of Oxford, Oxford, United Kingdom; 11 Department of Genome Sciences, University of Washington, Seattle, *Washington*, United States of America; University of Notre Dame, UNITED STATES

## Abstract

**Background:**

Sulfadoxine-pyrimethamine (SP) is recommended in Africa in several antimalarial preventive regimens including Intermittent Preventive Treatment in pregnant women (IPTp), Intermittent Preventive Treatment in infants (IPTi) and Seasonal Malaria Chemoprevention (SMC). The effectiveness of SP-based preventive treatments are threatened in areas where *Plasmodium falciparum* resistance to SP is high. The prevalence of mutations in the dihydropteroate synthase gene (*pfdhps*) can be used to monitor SP effectiveness. IPTi-SP is recommended only in areas where the prevalence of the *pfdhps*540E mutation is below 50%. It has also been suggested that IPTp-SP does not have a protective effect in areas where the *pfdhps*581G mutation, exceeds 10%. However, *pfdhps* mutation prevalence data in Africa are extremely heterogenous and scattered, with data completely missing from many areas.

**Methods and findings:**

The WWARN SP Molecular Surveyor database was designed to summarize dihydrofolate reductase (*pfdhfr)* and *pfdhps* gene mutation prevalence data. In this paper, *pfdhps* mutation prevalence data was used to generate continuous spatiotemporal surface maps of the estimated prevalence of the SP resistance markers *pfdhps*437G, *pfdhps*540E, and *pfdhps*581G in Africa from 1990 to 2020 using a geostatistical model, with a Bayesian inference framework to estimate uncertainty. The maps of estimated prevalence show an expansion of the *pfdhps*437G mutations across the entire continent over the last three decades. The *pfdhps*540E mutation emerged from limited foci in East Africa to currently exceeding 50% estimated prevalence in most of East and South East Africa. *pfdhps*540E distribution is expanding at low or moderate prevalence in central Africa and a predicted focus in West Africa. Although the *pfdhps*581G mutation spread from one focus in East Africa in 2000, to exceeding 10% estimated prevalence in several foci in 2010, the predicted distribution of the marker did not expand in 2020, however our analysis indicated high uncertainty in areas where *pfdhps*581G is present. Uncertainty was higher in spatial regions where the prevalence of a marker is intermediate or where prevalence is changing over time.

**Conclusions:**

The WWARN SP Molecular Surveyor database and a set of continuous spatiotemporal surface maps were built to provide users with standardized, current information on resistance marker distribution and prevalence estimates. According to the maps, the high prevalence of *pfdhps*540E mutation was to date restricted to East and South East Africa, which is reassuring for continued use of IPTi and SMC in West Africa, but continuous monitoring is needed as the *pfdhps*540E distribution is expanding. Several foci where *pfdhps*581G prevalence exceeded 10% were identified. More data on the *pfdhps*581G distribution in these areas needs to be collected to guide IPTp-SP recommendations. Prevalence and uncertainty maps can be utilized together to strategically identify sites where increased surveillance can be most informative. This study combines a molecular marker database and predictive modelling to highlight areas of concern, which can be used to support decisions in public health, highlight knowledge gaps in certain regions, and guide future research.

## Author summary

Despite great success in reducing death and illness from malaria over the last 20 years, the disease is still one of the main leading causes of death in low-income countries with estimated 229 million cases and 409,000 deaths annually. One of the main obstacles in malaria control is the development and spread of drug resistance. Several intermittent preventive treatments depend on the efficacy of the antimalarial drug sulfadoxine-pyrimethamine (SP); Intermittent Preventive Treatment in pregnant women (IPTp), Intermittent Preventive Treatment in infants (IPTi) and Seasonal Malaria Chemoprevention (SMC). Mutations in the dihydropteroate synthase gene (*pfdhps*) can cause resistance to SP treatment. In this paper, we use *pfdhps* mutation prevalence data to generate continuous spatiotemporal surface maps of the estimated prevalence of the SP resistance markers in Africa from 1990 to 2020 using a Bayesian geostatistical model. These predictive maps provide much needed insight about where SP can be used as part of preventive treatments. Spatial information on the spread of antimalarial resistance is critical for health organizations to prioritize surveillance measures, and plan control and elimination efforts.

## Introduction

Antimalarial drugs are essential tools for the control and elimination of malaria. Resistance to all currently available antimalarials, including the pivotal artemisinin derivatives, has been confirmed in the Greater Mekong Sub-region, with worrying signals of spread to or emergence in India [1–3], and recently, presence of de novo mutations in portions of the *Plasmodium falciparum* gene encoding kelch (K13)–propeller domains in Rwanda, Uganda, Eritrea and Ghana which can mediate artemisinin resistance [4–6].This situation is unfortunately reminiscent of the emergence and spread of parasites resistant to chloroquine and later sulfadoxine–pyrimethamine (SP) that resulted in dramatic increases in malaria-related morbidity and mortality across sub-Saharan Africa [7].

SP was used as a first-line treatment, alone or in combination with amodiaquine or chloroquine, for uncomplicated falciparum malaria in many sub-Saharan countries from the mid to late 1990s. Due to the rapid spread of SP-resistant parasites, SP was discontinued as recommended treatment in the early 2000s when artemisinin-based combinations became available and were progressively recommended as first-line treatment [8]. SP is currently recommended in Africa in several antimalarial preventive regimens including Intermittent Preventive Treatment in pregnant women (IPTp), Intermittent Preventive Treatment in infants (IPTi) and Seasonal Malaria Chemoprevention (SMC).

Point mutations in the dihydrofolate reductase gene (*pfdhfr*) at codons N51, C59, S108, and I164 confer resistance to pyrimethamine while point mutations in the dihydropteroate synthase gene (*pfdhps*) in codons S436, A437, K540, A581, and A613 are associated with resistance to sulfadoxine in the *P*. *falciparum* parasite. Molecular studies have shown that the triple mutant haplotype in *pfdhfr* (S108N, C59R, N51I) in combination with a double mutant haplotype of *pfdhps* (A437G, K540E), known as the quintuple mutant haplotype, is strongly associated with an increased risk of SP treatment failure in Africa [9, 10]. High prevalence of two single nucleotide polymorphisms, *pfdhfr*59R and *pfdhps*540E, could act as simpler surrogate markers for the quintuple mutant genotype and predict risk of SP treatment failure [9, 11]. In sub-Saharan Africa *pfdhfr*59R prevalence is higher than 75% in 73 out of 87 sites (filter: data collection 2010–2020, sample size > 49) [12]. As the prevalence of the triple mutant *pfdhfr* haplotype is very high across Africa, the prevalence of *pfdhps*540E alone could be used as a surrogate marker for the quintuple haplotype and which is highly resistant to SP. The additional mutation *pfdhps*A581G increases SP resistance modestly *in vitro* [13] and is associated with increased risk of SP treatment failure [14].

Currently SP is mainly used for IPTp to reduce maternal malaria episodes, maternal anaemia, low birth weight, and neonatal mortality. IPTp-SP is recommended by WHO for all pregnant women living in areas of moderate-to-high malaria transmission in Africa [15, 16]. In addition, SP is recommended by WHO for IPTi, where a full course of SP is administered to infants, independently of presence of parasitemia, to reduce the malaria burden [17]. It is recommended that IPTi-SP is not implemented in areas where *pfdhps*540E exceeds 50%. Monitoring of prevalence of other molecular markers for SP resistance is also recommended, in particular *pfdhps*A581G, however they are not yet used to guide IPTi policy [18].

The effectiveness of IPTp in sub-Saharan Africa is threatened in areas where *P*. *falciparum* is highly resistant to SP. The sextuple mutant haplotype (*pfdhfr* S108N, C59R, N51I in combination with *pfdhps* A437G, K540E, A581G), was associated with increased risk of *P*. *falciparum* infection, and higher parasitaemia in pregnant women receiving IPTp-SP and a more intense placental inflammation in Malawi and Tanzania [19, 20]. An initial meta-analysis based on five studies, concluded that IPTp-SP did not reduce the risk of low birth weight in infants in studies in East Africa where *pfdhps*540E exceeded 50% [21]. In a recent, comprehensive analysis, it was demonstrated that IPTp-SP mediated reductions in the risk of low birthweight decline with increasing *pfdhps*540E prevalence. However, even in areas where *pfdhps*540E prevalence exceeds 90%, modest reductions in risk of low birth weight remain, if *pfdhps*581G prevalence is below 10%. The point mutation *pfdhps*581G can serve as a proxy for the sextuple mutant haplotype. Concerningly, in regions where prevalance of the sextuple mutant exceeded 10%, IPTp-SP no longer protected newborns against low birth weight. The estimated pooled *pfdhps*581G prevalence was 37% in the aggregated analyses of studies in these regions [22].

SP+AQ is currently recommended for SMC in countries in the Sahel sub-region of Africa with intense seasonal malaria, but not in East and Southern Africa due to spread of the highly SP-resistant quintuple mutant parasite [23], which can be monitored by assessing the prevalence of *pfdhps*540E [24]. The introduction of SP+AQ SMC has been accompanied by a local increase in SP resistance marker prevalence. In Southern Mali, the prevalence of *pfdhps*540E, and the quintuple mutant haplotype significantly increased in children after receiving SP+AQ SMC, however the chemoprevention was still effective [25]. In a large study conducted in seven countries in West and Central Africa, the prevalence of *pfdhps*540E increased in *P*. *falciparum* infected children who did not receive SMC but lived in areas where SP+AQ SMC was deployed [26].

Understanding the spatio-temporal distribution and prevalence of *pfdhps* gene mutations across Africa is essential to inform effective targeting of SP for IPTp, IPTi and SMC. Spatiotemporal models can support the monitoring of drug resistance and appropriate targeting of the preventive strategies IPTp, IPTi and SMC. The aims of this study were first to update the previously published database with more recent data on the prevalence of markers of SP resistance and second, to build a spatiotemporal model to provide an up-to-date picture on the distribution of *pfdhps*437G, *pfdhps*540E, and *pfdhps*581G mutations, the markers relevant for monitoring the effectiveness of SP-IPTp, IPTi and potentially SMC.

## Methods

### Data summary

In this study the drug-resistance marker prevalence was analysed. This variable refers the proportion of individual patient blood samples that test positive for a given mutation or combination of mutations out of the tested malaria infected individuals. Data on the prevalence of the *pfdhps*437G, *pfdhps*540E and *pfdhps*581G mutations were extracted from articles published between January 1997 and April 2020. These studies covered information on marker prevalence from samples collected from 1978 to 2018. The data were extracted from the following sources; 1) the Drug Resistance Maps database (publications 1997–2011), 2) the WorldWide Antimalarial Resistance Network (WWARN) SP Molecular Surveyor database (publications 2011–2020) and 3) data shared with the WWARN repository. From these sources, data on *pfdhps*437G, *pfdhps*540E and *pfdhps*581G mutation prevalence, year of sample collection, location of collection and publication details were extracted. Some tested isolates contain parasites with both wildtype and mutant alleles. To account for this, the prevalence of a mutation was defined as the number of samples containing the mutant allele, either pure, or mixed with the wild-type allele, divided by the total number of samples tested. This information was used to inform the final model, further described below, where prevalence was estimated by marker each year from 1990 to 2020.

### Drug resistance maps database

Data from the Drug Resistance Maps database was used as described previously [27, 28]. Briefly, a literature search was conducted to identify articles published from 1997 to 2011 with data on prevalence of *pfdhfr* and *pfdhps* mutations in Africa. Study site, study year and the proportion of isolates with a particular mutation were recorded in a database [27, 28]. All data on prevalence of *pfdhps*437G, *pfdhps*540E and *pfdhps*581G mutations, study site and year were extracted from the database, for the model outputs.

### WWARN SP molecular surveyor database

The WWARN SP Molecular Surveyor database and visualization tool was created to summarize data on SP resistance markers in the *pfdhfr* and *pfdhps* genes, derived from publications and studies shared with the WWARN. To identify appropriate publications, a literature search was conducted in PubMed with the search terms ‘malaria AND (*dhfr* OR *dhps* OR *pfdhfr* OR *pfdhps OR “molecular marker” OR “molecular markers”*)’. Inclusion criteria were; 1) at least one *P*. *falciparum pfdhfr* or *pfdhps* genotype or haplotype, 2) primary data source, 3) baseline/pre-treatment isolates, and 4) meta-data on collection of samples including the year and location (at least on country level) of sample collection. Inclusion and exclusion criteria and the standardized data extraction process are described in detail in S1 Text. Details of the extraction process can be found in the S2 Text. Publications from 2011–2020 were included in the WWARN SP Molecular Surveyor database.

The current maps include data from samples collected during therapeutic efficacy studies and routine surveillance of antimalarial efficacy in Mpumalanga, South Africa, 2016–2018 using malaria-positive RDTs collected from various primary healthcare facilities within the malaria-endemic districts [29, 30]. Differences in study design are not explicitly accounted for in the modelling.

For the final model outputs, data on location, year and mutation prevalence were extracted from the SP Molecular Surveyor database. Studies of prevalence of *pfdhps*437G, *pfdhps*540E and *pfdhps*581G mutations from the African continent published from 1 January 2011 to 21 April 2020 were included in the analysis. To avoid duplication, studies already entered in the Drug Resistance Maps database, described above, were excluded from the selection.

### Geostatistical modelling of molecular markers

In this paper, we used a geostatistical model to generate a continuous spatio-temporal surface to estimate the prevalence of *pfdhps*437G, *pfdhps*540E and *pfdhps*581G markers associated with SP resistance. The *pfdhps* data, which were only available at discrete study locations and times were used to predict the prevalence of these three markers across Africa from 1990–2020. In this way, the model output provided insight into the spatiotemporal spread of resistance in a way that the discrete data points alone cannot provide. A study site, in this context, refers to samples collected in a specific location and year within a study. The geostatistical model included as a covariate the *P*. *falciparum* transmission intensity available from 2010–2017, as estimated by the spatiotemporal models developed by the Malaria Atlas Project (MAP) [31]. For years before 2010, we use the 2010 transmission intensity and for years after 2017, we use the 2017 transmission intensity. Full details of the geostatistical model are provided in S2 Text and a conditional dependency schematic for the geostatistical model in S1 Fig.

The statistical methodology follows two stages to allow for spatiotemporal prediction of the molecular marker prevalences, which are outlined here briefly (see S2 Text for details). Firstly, based on the observed data, the posterior distribution of model parameters was estimated using a Bayesian inference framework. Secondly, given the model parameters from the first stage, marker prevalence was predicted on a 5 x 5 km grid within the *P*. *falciparum* spatial limits of Africa (defined by MAP) for each year from 1990 to 2020. For each location, a distribution of prevalences was drawn from the posterior predictive distribution and summarized using the median statistic to create a single continuous surface. The standard deviation surface of the posterior predictive distribution was presented alongside the median maps as a summary of the associated uncertainty in the predictions at each location/time. This process was repeated separately for each of the three molecular markers. That is, we present the *posterior predictive median* as an estimate of marker prevalence and the *posterior predictive standard deviation* as a measure of uncertainty of the prevalence in each 5 x 5 km pixel within the *P*. *falciparum* spatial limits of Africa (defined by MAP) for each year from 1990 to 2020.

Model validity was assessed to ensure sound interpretation of the model output. For each marker, the corresponding dataset was divided into ten subsets selected at random. Each of the ten subsets of data was treated as a validation set to test the model’s predictive ability by running the model with the subset withheld. The ability of the model to predict marker prevalences at the locations/times in that subset was tested against the actual withheld prevalence data. The predictive results for each of the ten subsets of data were pooled, so that each datapoint had an associated predictive validation distribution from which model validity was assessed (see S2 Text).

### Visualization of observed data

Data visulizations of the observed prevalence of the *pfdhps*581G mutation in sites exceeding 10% prevalence in the results was performed using Tableau (Tableau Software, Seattle, WA).

## Results

### Data summary

Data on the prevalence of *pfdhps*437G, *pfdhps*540E and *pfdhps*581G mutations from 201 studies published between 1 January 1997 and 21 April 2020 were included in the analysis. These studies covered data from samples collected from 1978 to 2018. In total, 1404 data points were analysed for the three mutations Table 1. Each data point refers to one study location per year per study. The full list of included studies can be found in S1 Data.

**Table 1:** Number of data points by data source and gene locus.

Locus	Drug Resistance Maps	Molecular Surveyor	WWARN repository
*pfdhps*437	229	247	39
*pfdhps*540	237	255	43
*pfdhps*581	121	195	38

The global prevalence of the studied *pfdhps* markers can be visualized online with the WWARN SP Molecular Surveyor, along with *pfdhfr* markers. The WWARN SP Molecular Surveyor [12] is regularly updated with data from recent publications and data shared with WWARN.

**Fig 1:**
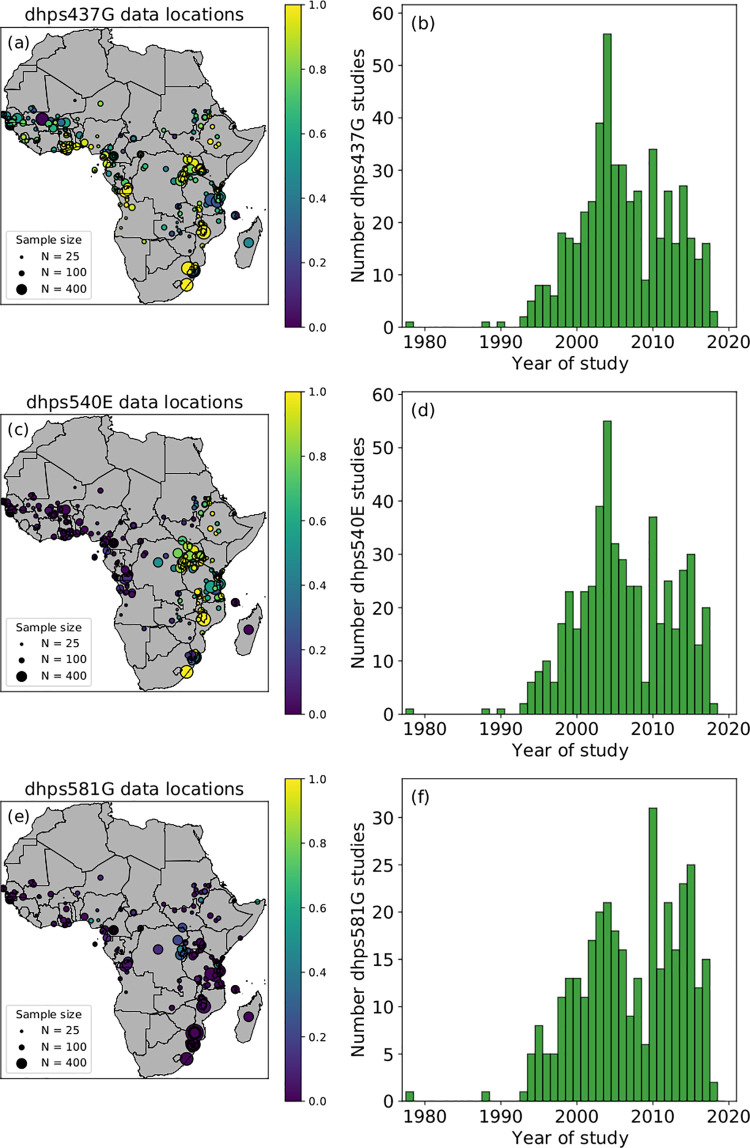
Spatial locations and *pfdhps* mutation prevalence from collected data. Summary of the spatial locations of the collected data and the prevalence for *pfdhps*437G (a), *pfdhps*540E (c) and *pfdhps*581G (e) across the African continent and the number of study sites per year during the time period 1980–2020 for *pfdhps*437G (b), *pfdhps*540E (d) and *pfdhps*581G (f). In (a), (c) and (e), the size of the dots is proportional to the study sample size and the colour is representative of the observed marker prevalence. National shapefiles were obtained from the Malaria Atlas Project (MAP; https://malariaatlas.org/) under their open access policy (https://malariaatlas.org/open-access-policy/) and no changes were made.

A summary of the location of the study sites, study sample size, prevalence and study sites per year of the data used in the mathematical modelling for *pfdhps*437G, *pfdhps*540E and *pfdhps*581G is shown in Table 2 and Fig 1. A substantial increase in median prevalence of the three markers was observed over the decades.

**Table 2:** Summary of the *pfdhps* marker data used in the mathematical models, by sample collection: 1978–1998, 1999–2008 and 2009–2018.

Marker	No. study sites	Median prevalence (Q1, Q3)	Year range sample collection*	Median sample size (Q1, Q3)
*pfdhps437G*	50	0.22 (0.08. 0.41)	1978–1998	46 (32, 68)
	286	0.67 (0.38, 0.90)	1999–2008	76 (40, 139)
	178	0.87 (0.67 0.99)	2009–2018	75 (31, 117)
*pfdhps540E*	52	0.06 (0.01 0.32)	1978–1998	49 (33, 71)
	289	0.16 (0.01 0.70)	1999–2008	75 (41, 135)
	193	0.22 (0.01, 0.92)	2009–2018	81 (33, 129)
*pfdhps581G*	37	0.01 (0.01, 0.01)	1978–1998	76 (37, 129)
	151	0.01 (0.01, 0.01)	1999–2008	82 (46 152)
	165	0.02 (0.01, 0.10)	2009–2018	81 (37, 126)

The reported year refers to the year of sample collection (rather than year of publication). Q1 and Q3 are the first and third quantile, respectively. * Note that the articles published 1997–2020 included data collected 1978–2018.

S1, S2 and S3 Videos show the time course of data collection for *pfdhps*437G, *pfdhps*540sE and *pfdhps*581G, respectively, over the period of 1990 to 2020. In the videos, the data visualized in each year show studies conducted before or during the year associated with the map.

### Geostatistical model

Continuous predictive maps for each of the three molecular markers were generated over the time period of 1990 to 2020 within the *P*. *falciparum* spatial limits of Africa, using the data shown in Fig 1. The construction of a statistical model provides two key advantages over the raw data: (1) there are many locations in space and time without data available where predictions are informative (in Fig 1, all data across any time point overlaid on the same spatial map) and (2) our model allows quantification of uncertainty in estimates which the raw data alone do not allow (consider two studies at the same space-time location).

**Fig 2:**
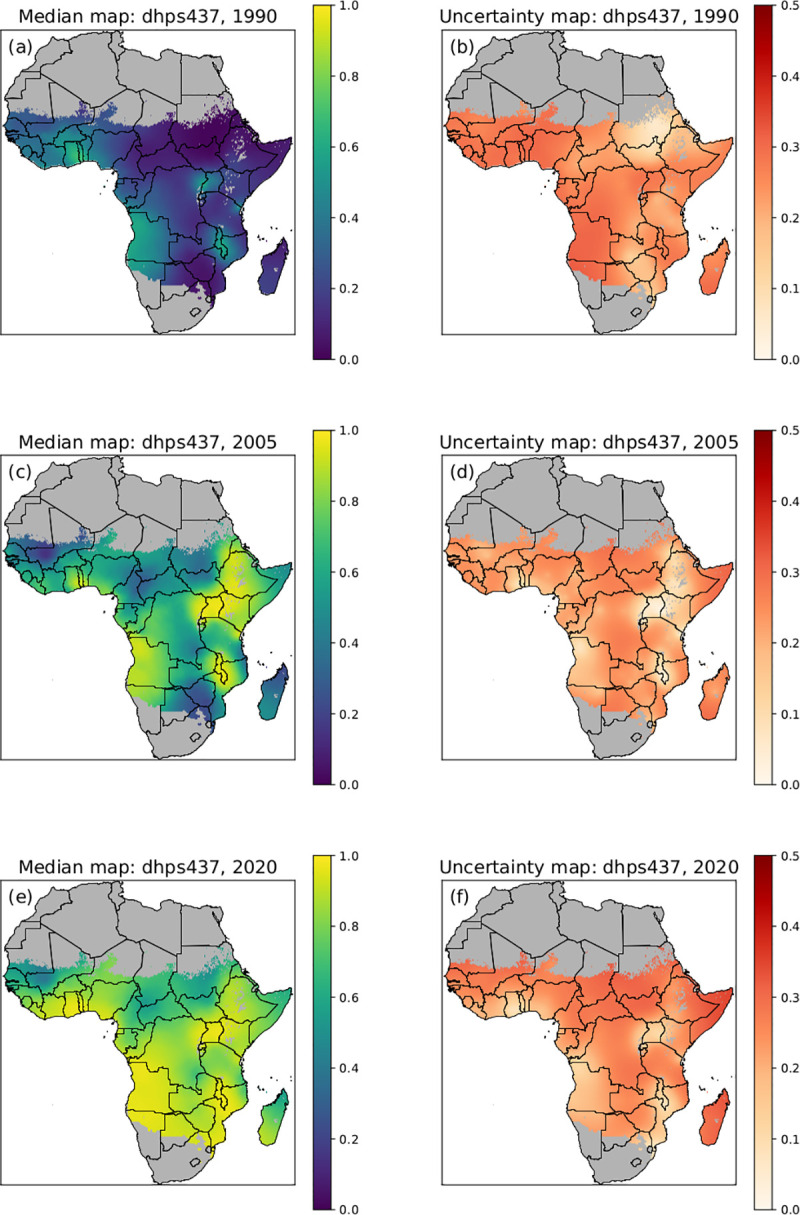
Posterior predictive median prevalence of *pfdhps*437G. Posterior predictive median prevalence of *pfdhps*437G in 1990 (a), 2005 (c) and 2020 (e). Associated standard deviations (uncertainty) for *pfdhps*437G posterior predictions in 1990 (b), 2005 (d) and 2020 (f). A low standard deviation (lighter colour) indicates low uncertainty and high confidence in the model. National shapefiles were obtained from the Malaria Atlas Project (MAP; https://malariaatlas.org/) under their open access policy (https://malariaatlas.org/open-access-policy/) and no changes were made.

First the predicted prevalence of the *pfdhps*437G marker was examined (Fig 2). The model enables us to make predictions in regions in space and time where there are no data, by drawing on the existing data that are available. However, generally speaking, using limited data and/or studies with smaller sample sizes will lead to higher levels of uncertainty. In 1990 *pfdhps*437G predicted prevalence was low throughout the continent, except for a few isolated locations in East and West Africa. By 2005, the prevalence of the *pfdhps*437G mutation was predicted to be significantly higher in locations in East and West Africa, and by 2020, the predicted *pfdhps*437G marker levels were high over the majority of the continent. The associated uncertainty maps showed moderate uncertainty about the predictions over most of the continent but was lower in regions of East and South Africa with higher *pfdhps*437G prevalence.

**Fig 3:**
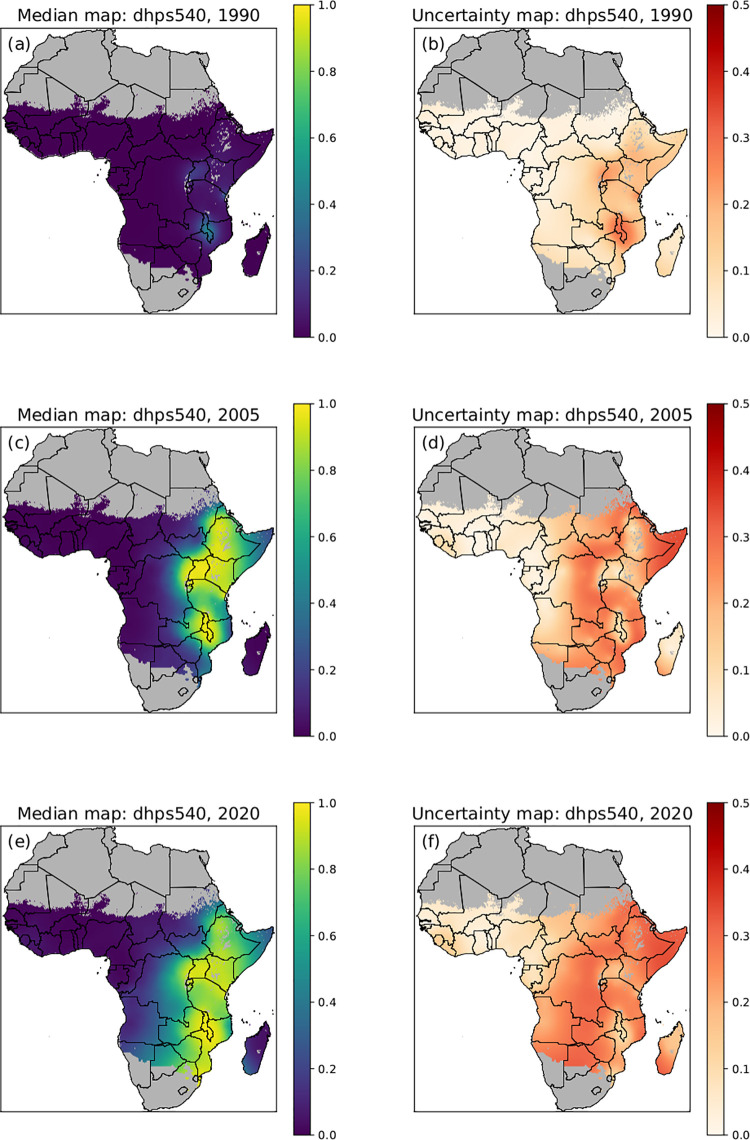
Posterior predictive median prevalence of *pfdhps*540E. Posterior predictive median prevalence of *pfdhps*540E in 1990 (a), 2005 (c) and 2020 (e). Associated standard deviations (uncertainty) for *pfdhps*540E posterior predictions in 1990 (b), 2005 (d) and 2020 (f). A low standard deviation (lighter colour) indicates low uncertainty and high confidence in the model. National shapefiles were obtained from the Malaria Atlas Project (MAP; https://malariaatlas.org/) under their open access policy (https://malariaatlas.org/open-access-policy/) and no changes were made.

Consistent with previous mathematical modelling [27] the median of the posterior predictive distribution of *pfdhps*540E was near zero over the entire continent in 1990 with the exception of a few ‘hotspots’ in East Africa (Fig 3). The uncertainty map showed that there was high confidence in these model results, but less so at the hotspots. From 1990 to 2005, there was significant spread of the *pfdhps*540E mutation in East Africa (but not in the west) and similarly, from 2005 to 2020 there was further spread of *pfdhps*540E mutations in the East and South East. The associated uncertainty maps show that there is increasing uncertainty in the predictions from 1990 to 2020, especially in regions of temporal change from low to high marker prevalence.

**Fig 4:**
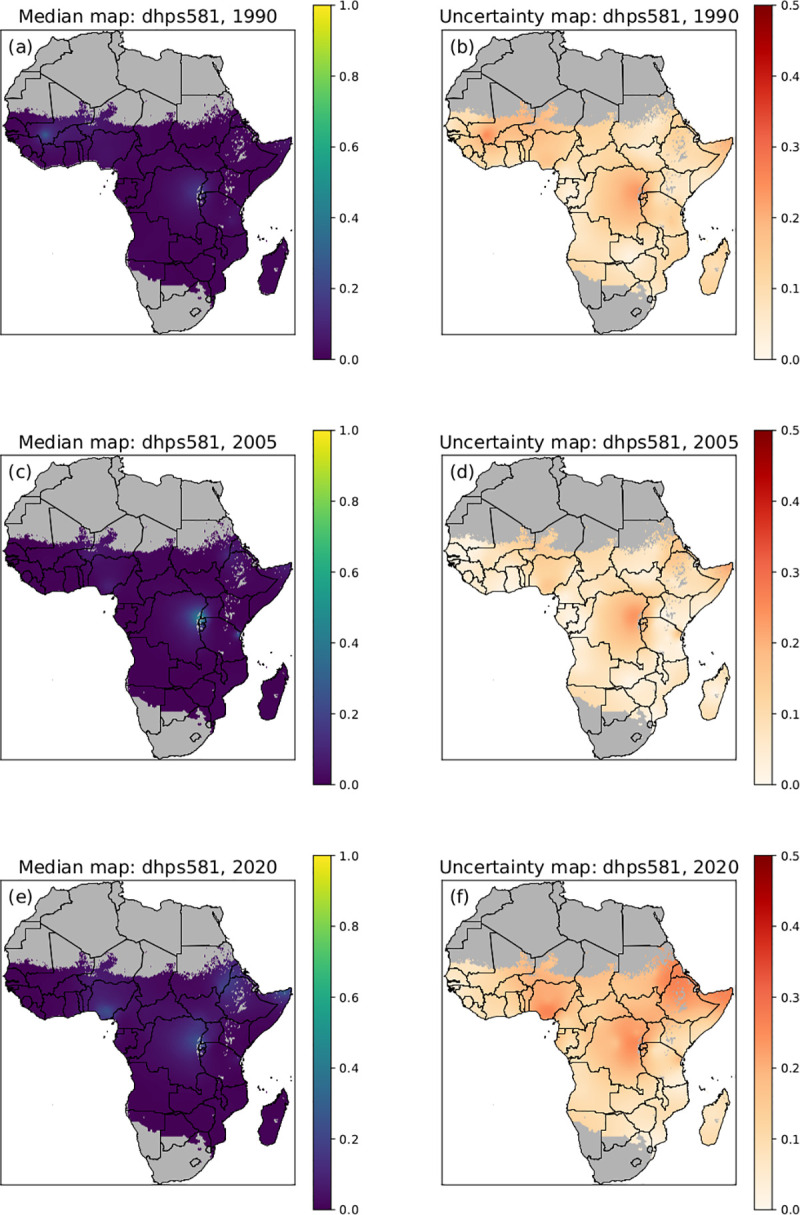
Posterior predictive median prevalence of *pfdhps*581G. Posterior predictive median prevalence of *pfdhps*581G in 1990 (a), 2005 (c) and 2020 (e). Associated standard deviations (uncertainty) for *pfdhps*581G posterior predictions in 1990 (b), 2005 (d) and 2020 (f). A low standard deviation (lighter colour) indicates low uncertainty and high confidence in the model. National shapefiles were obtained from the Malaria Atlas Project (MAP; https://malariaatlas.org/) under their open access policy (https://malariaatlas.org/open-access-policy/) and no changes were made.

In Fig 4 the model results for *pfdhps*581G mutation prevalence in 1990, 2005 and 2020 are shown. The predicted prevalence for *pfdhps*581G is near zero every year and the uncertainty in these predictions is consistently low (i.e., high confidence). There are some locations where the predicted marker levels are slightly above zero with increasing prevalence over time in central Africa in Rwanda and along Rwanda border in Uganda, Tanzania and DRC, Nigeria and Horn of Africa, but these are associated with higher uncertainty. S4, S5 and S6 Videos show the median of the posterior predictive distribution for *pfdhps*437G, *pfdhps*540E and *pfdhps*581G mutation prevalence respectively over 1990 to 2020.

**Fig 5:**
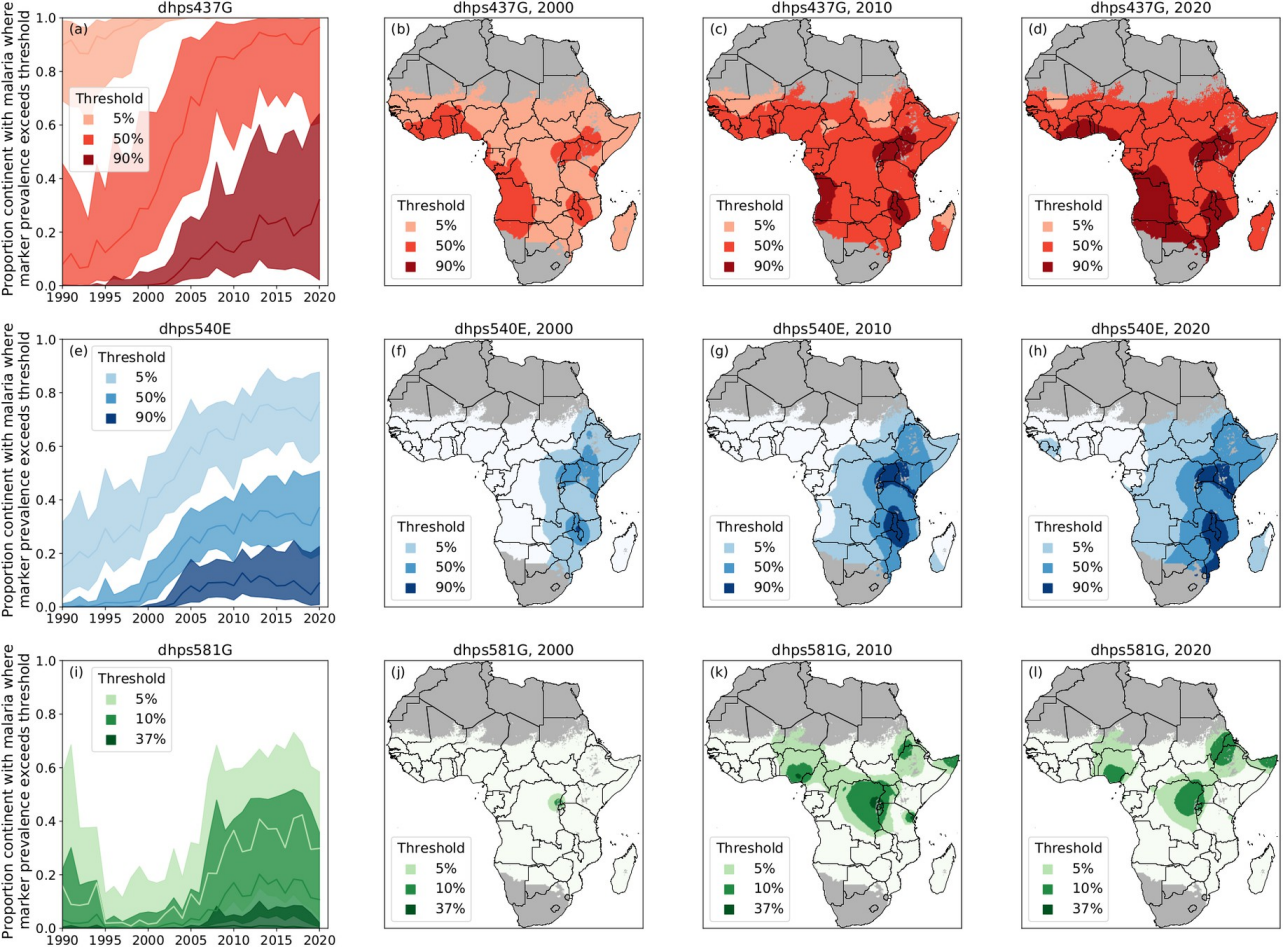
Predicted proportion of Africa that exceeds specific prevalence thresholds. The proportion of the continent within the *Pf* spatial limits of Africa with *pfdhps*437G (a), *pfdhps*540E (e) and *pfdhps*581G (i) prevalence exceeding relevant thresholds over the time period of 1990 to 2020. The median estimates are shown in the solid-colored lines and the associated uncertainty (50% credible intervals) in the shaded regions. The predicted area with prevalence exceeding relevant thresholds shown in three shades, based on median predictions, for *pfdhps*437G (red), *pfdhps*540E (blue) and *pfdhps*581G (green) in 2000 ((b), (f), (j)), in 2010 ((c), (g), (k)), and 2020 ((d), (h), (l)). The predictive proportions displayed for *pfdhps*437G (red) and *pfdhps*540E (blue) are 90%, 50% and 5%. For *pfdhps*581G (green), present in lower prevalence, the proportions displayed are 37%, 10% and 5%. National shapefiles were obtained from the Malaria Atlas Project (MAP; https://malariaatlas.org/) under their open access policy (https://malariaatlas.org/open-access-policy/) and no changes were made.

We further examined the temporal trends of the predicted proportion of Africa (within the *P*.*falciparum* spatial limits) that contained infections with the studied mutations. An expansion of the *pfdhps*437G mutation over the last three decades awas observed and in 2020 high prevalence of *pfdhps*437G (50% and higher) was predicted in almost all of Africa (Fig 5a–5d).

*pfdhps540E* distribution (in blue) at therapeutically relevant prevalence thresholds was examined; 90% where IPTp is suggested to have only limited effect and 50% where IPTi is no longer recommended. The *pfdhps*540E mutation, the marker for the quintuple mutation, emerged from limited foci in East Africa to currently exceeding 50% estimated prevalence in most of East and South Africa and is present in low to moderate prevalence in Central Africa. Interestingly, in West Africa *pfdhps*540E remains absent or rare (Fig 5e–5h).

Distribution of *pfdhps*581G (in green) was analyzed at relevant predicted prevalence thresholds; 37% and 10%) when the IPTp has been suggested to lose its effect. The *pfdhps*581G distribution expanded from one main focus in 2000 to four foci in 2010 in: 1) Rwanda/East DRC/South West Uganda/North West Tanzania, 2) Nigeria and 3) North East Tanzania, and, 4) Sudan/Eritrea/Ethiopia, where *pfdhps*581G predicted prevalence exceeded 10%. *pfdhps*581G predicted prevalence seems to decrease slightly predicted between 2010 and 2020 and high uncertainty was notes in areas where *pfdhps*581G was present (Figs 4 and 5i–5l).

**Fig 6:**
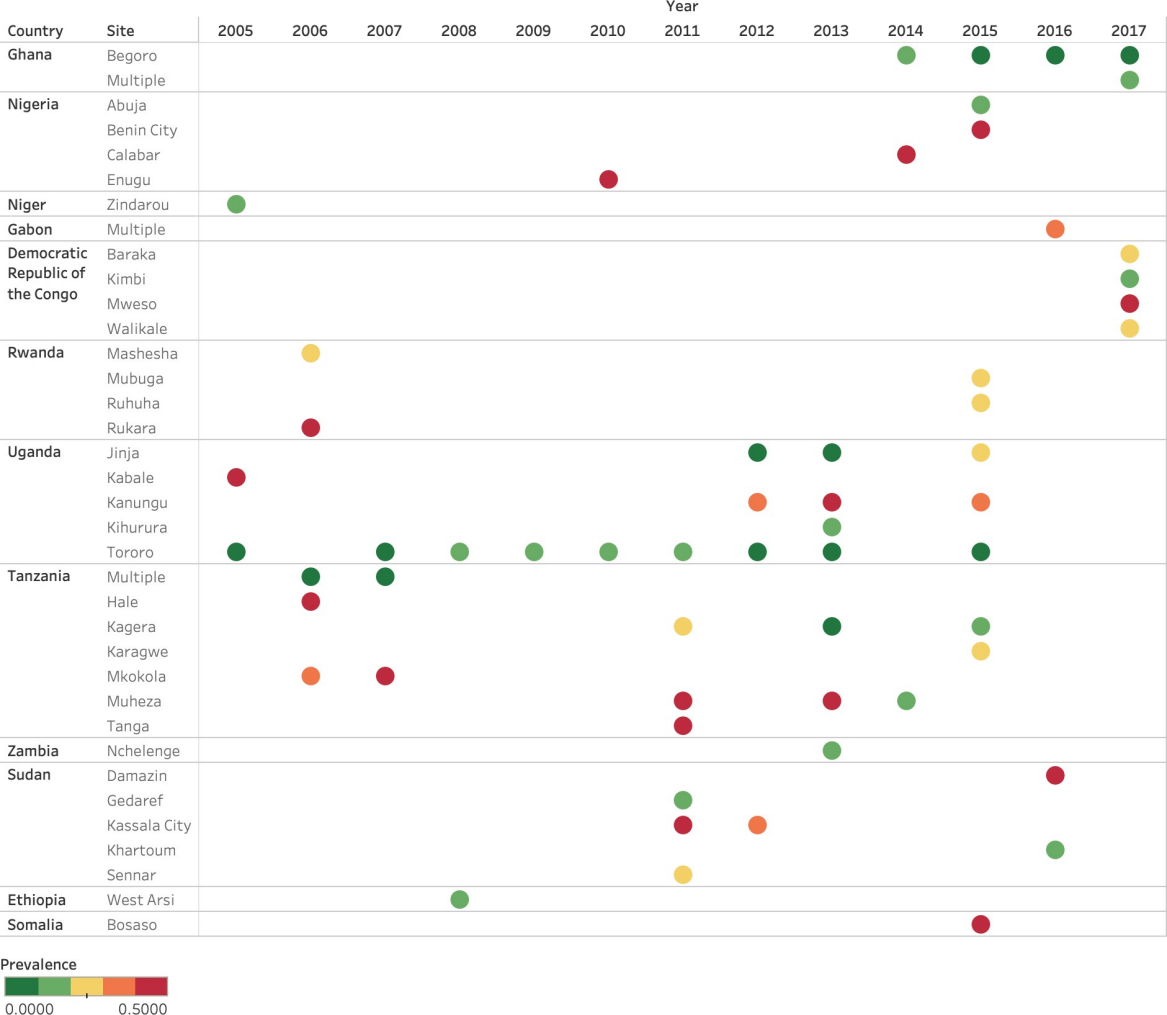
Locations where *pfdhps*581G prevalence has exceeded 10%. Observed prevalence of *pfdhps*581G is displayed for all years in sites where *pfdhps*581G prevalence was >10% at least one year, from 2005 to 2018.

**Fig 7:**
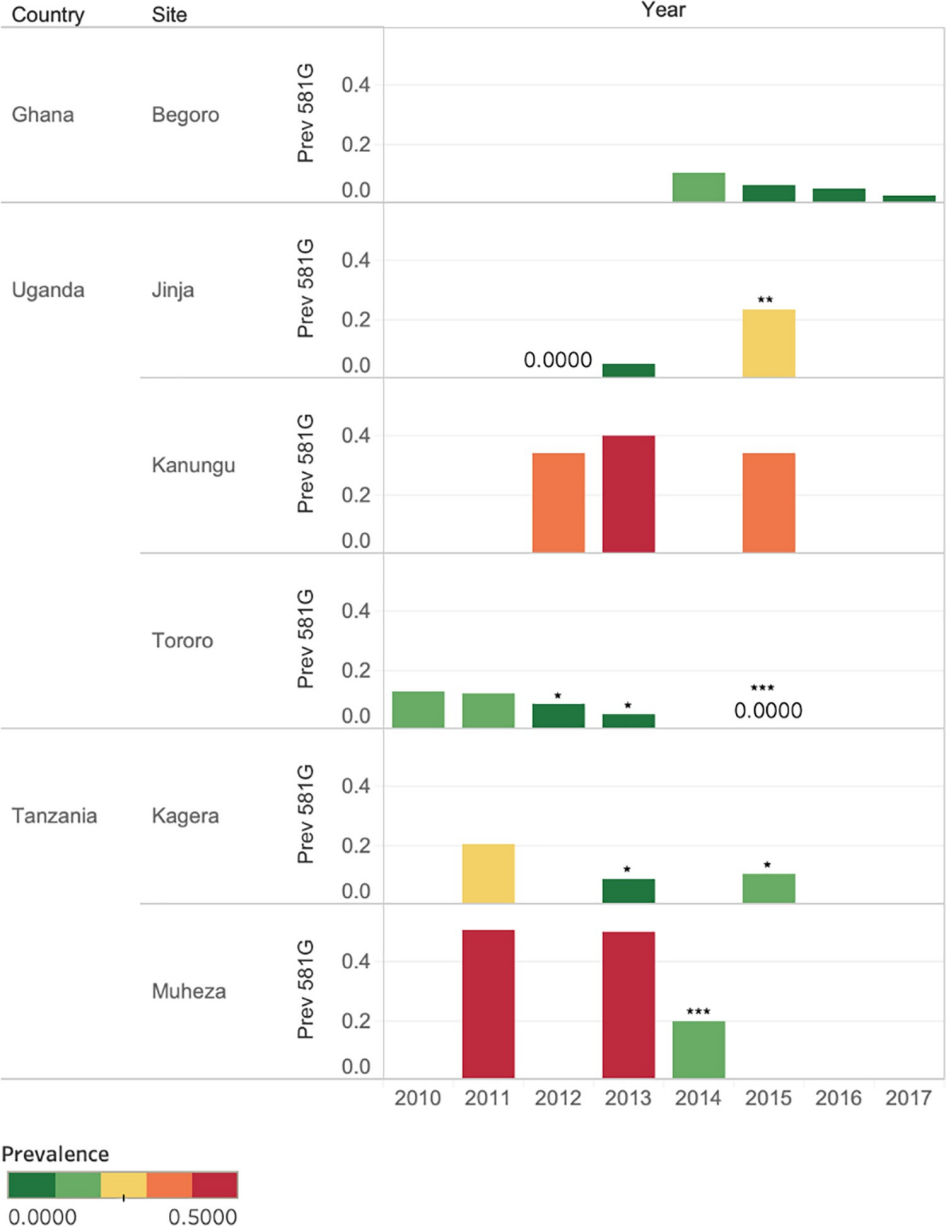
Locations where *pfdhps*581G prevalence has exceeded 10% and data from three years are available. Observed prevalence of *pfdhps*581G is displayed for all years in sites where *pfdhps*581G prevalence was >10% at least one year and data from at least three years were available, between 2010 to 2018. A statistically significant change in *pfdhps*581G prevalence in comparison to the first assessed year using Fisher’s exact test is displayed as * (P ≤ 0.05), ** (P ≤ 0.01) or *** (P ≤ 0.001).

Could the slightly decreased *pfdhps*581G prevalence observed in the model output be explained by the absence of data from study sites with high *pfdhps*581G prevalence or by an actual decrease in *pfdhps*581G prevalence? This question was further investigated in the observed data set in Fig 6. The dynamics of *pfdhps*581G prevalence over time from 2006–2018 was examined in study sites where *pfdhps*581G prevalence was > 10% in at least one year. Prevalence of *pfdhps*581G was observed to be higher than 37% in at least one site and year, between 2010 and 2017 in studies conducted in Nigeria, Democratic Republic of Congo, Uganda, Tanzania, Sudan, and Somalia, and in 2006 in Rwanda. For many of these sites, data was only available from one year, hindering trend analysis. To determine how *pfdhps*581G prevalence was changing over time, sites that had data from at least three years were further examined in Fig 7. A statistically significant increase in *pfdhps*581G prevalence was observed in Jinja, Uganda and a decrease was observed in Muheza and Kagera in Tanzania and Tororo Uganda, while Kanunga, Uganda showed a continuously high prevalence and Begoro, Ghana showed a continuously low prevalence (Fig 7, S2 Data). A decrease in *pfdhps*581G prevalence was observed in several sites, and could partly explain the *pfdhps*581G decrease in the model output in some areas, but not all. Another main determinant was likely an absence of data for sites where high prevalence was previously observed (e.g., all sites in Rwanda and Nigeria, Fig 6), which is reflected in the high level of uncertainty which accompanies the presence of *pfdhps*581G (Fig 4).

To further understand the lag time between data collection and publication, the time between the collection of the most recent data in the publication and the publication year was evaluated. The mean time to publication after data collection with a 95% confidence level was 3.66 ± 0.42 years and the median time was 3 years (range 0–39 years).

### Model validation

By rerunning the mathematical model 10 times, each time with a different 10% of the data withheld, the model validity was assessed. Table 3 shows the correlation coefficient, mean error and mean absolute error between the observed and median predicted marker prevalence at each study site (e.g., space-time location) for each of the three molecular markers. Overall, there is good agreement between the observed and predicted prevalences, with the mean error (measure of bias) lowest for *pfdhps*540E and the mean absolute error (measure of average discrepancy) lowest for *pfdhps*581G. Since the observed values of *pfdhps*581G are overall lower than the other markers it follows that the mean absolute error is small. S2 Fig shows validation results for the *pfdhps*540E mutation. The scatterplot of the predicted median prevalence from the validation models and observed prevalence (S2a Fig) provided further evidence of strong agreement between the observed and predicted *pfdhps*540E prevalences. S2b Fig shows a probability-probability plot of the fraction of *pfdhps*540E observations that fell within a predictive credible interval of a given size and indicates that the reliability of the credible intervals was strong, even for narrower credible intervals. S3 Fig and S4 Fig show the validation results for *pfdhps*437G and *pfdhps*581G, respectively, and indicate agreement between the observed and predicted prevalences (although not as strong as for *pfdhps*540E*)* as well as strong reliability of the credible intervals.

**Table 3:** Correlation coefficient, mean error and mean absolute error by molecular marker.

Marker	Correlation coefficient	Mean error	Mean absolute error
*pfdhps437*	0.837	1.902	11.868
*pfdhps540*	0.950	0.031	7.818
*pfdhps581*	0.824	-1.810	2.897

Summary of the correlation coefficient, mean error and mean absolute error between the observed marker prevalence and the predicted (median) prevalence at the space-time locations of the studies for each of the three molecular markers.

## Discussion

Continuous spatiotemporal surface maps of the estimated prevalence of the SP resistance markers *pfdhps*437G, *pfdhps*540E, and *pfdhps*581G in Africa between 1990 and 2020 were generated using a geostatistical model, with a Bayesian inference framework to estimate uncertainty. The newly generated maps show an expansion of the *pfdhps*437G mutations across the entire continent over the last three decades, with the *pfdhps*540E mutation emerging in different places in East Africa and spreading from the Horn of Africa to South Africa but remaining highly prevalent only in the East and South East African regions to date. Although the *pfdhps*581G mutation has emerged in various places across the continent, its prevalence remains, to date, relatively low. The information in the geostatistical model and spatiotemporal maps can be used to inform public health decision making and guide smarter selection of sites for data collection to further refine the available data sets.

### Why does SP resistance increase

Notwithstanding the limitations of the underlying data/model discussed below, the maps clearly indicate the broad trends of changes in marker prevalence over time and space. The spatiotemporal surface maps present the changing distribution of the *pfdhps*437G and *pfdhps*540E markers, associated with SP treatment failure [9], over the study period.

Although SP was discontinued as a first line antimalarial for uncomplicated *P*. *falciparum* malaria in the majority of African countries between 2001 and 2007 [8], the predicted *pfdhps*437G and *pfdhps*540E marker prevalence has continued to increase and the estimated area where *dhps*540E was present increased in East Africa (Figs 2, 3 and 5). Multiple factors may contribute to the continued high prevalence of *pfdhps*437G and *pfdhps*540E. First, these mutations may not affect parasite fitness. It has been suggested that the quintuple mutant haplotype incurs little or no fitness cost, as a high frequency of the mutation remained in Malawi despite apparent absence of strong SP drug pressure [32]. Second, SP is still being used throughout Africa, in IPTp, IPTi and SMC, and possibly as an informal treatment of uncomplicated malaria. IPTp-SP was recommended by WHO in 2004 [16], however implementation was slow. In 2014–2016, the proportion of women who took IPTp-SP in their most recent pregnancy in eight African countries was estimated to be 30% [33]. As observed in a study in Western Kenya, the increase in prevalence of the quintuple mutant genotype coincided with increased use of IPTp-SP [34]. IPTi-SP is recommended in countries with *pfdhps*540E prevalence below 50%, and after IPTi administration an increase in *pfdhps* mutation prevalence was observed in one study in Sierra Leone [35], but not in studies in Mali and Tanzania [36, 37]. SP continues to be used in combination with amodiaquine for SMC in children in the Sahel region in Africa, where the quintuple mutant genotype prevalence increased in children receiving SMC [38] and *pfdhps*540E has increased in areas where SMC has been deployed [26]. A recent meta-analysis across Africa showed that in 2015 only 19.7% of children with malaria received an ACT treatment [39], indicating that other treatment options, like SP, are likely to be still in use particularly by those who procure malaria treatments from informal drug vendors. Last, a similar drug to SP, cotrimoxazole (sulfamethoxazole/trimethoprim) is taken by many HIV positive individuals, maintaining at least some drug pressure on the parasite populations [8]. A limitation of this study is that it did not provide information on *pfdhps* haplotypes, but was restricted to the prevalence of *pfdhps* single genotypes due to limited number of studies that collected data on *pfdhps* haplotypes. Due to the particular distribution of the *pfdhps* mutations in Africa, single mutations can act as surrogate markers for haplotypes. *Pfdhps* mutations emergence across Africa on an already established background of mutant *pfdhfr* coincided with the first clinical treatment failure with SP in Africa in the 1990s [40]. In East Africa, the double mutant *pfdhps*437G and *pfdhps*540E together with *pfdhps*436S (SGE) emerged. In west Africa, the *pfdhps*437G substitution was found alone or together with *pfdhps*436S, and the SGE allele was rare or absent [40]. Clinical resistance to SP were closely associated with the quintuple haplotype (*pfdhfr* S108N, C59R, N51I in combination with *pfdhps* A437G, K540E) [9]. Therefore *pfdhps*540E can be used as a proxy for the quintuple haplotype. *pfdhps*581G mutation was observed to have emerged locally on *pfdhps*437G+ *pfdhps*540E backgrounds in several locations in Africa [41]. While single *pfdhps*540E or *pfdhps*581G mutations do occur independently, these strains are rare.*pfdhps* 581G, of interest for decreased effect of IPTp-SP, and *pfdhps*540E can be used as a proxy for the sextuple haplotype (*pfdhfr* S108N, C59R, N51I in combination with *pfdhps* A437G, K540E, A581G).

### Implications for SMC

In 2012 SP+AQ SMC was recommended to be used only in the Sahel region, due to high resistance to SP in East and Southern Africa, marked by high prevalence of *pfdhps*540E [24]. Our spatiotemporal maps demonstrate an increase of predicted prevalence of *pfdhps*540E in East and Southern Africa over time (Figs 1 and 3), particularly apparent in the display of the expanding areas of *pfdhps*540E prevalence (Fig 5). The predicted prevalence of *pfdhps*540E was consistently low (<5%) in West and Central Africa, however there were scattered sites such as Equatorial Guinea [42] and Nigeria [43] with higher observed prevalence of *pfdhps*540E (Fig 1). While general reasons for the increase of SP resistance were previously discussed, SP+AQ SMC use may also promote resistance. It has been demonstrated that SP+AQ SMC can select for *pfdhfr* and *pfdhps* SNPs in individuals that receive the chemoprophylaxis and that individuals that live in areas where SMC is used are more likely to be infected with a *pfdhps*540E-carrying parasite [26, 38]. In these studies, *pfdhps*540E prevalence was still low, and the increase did not seem to impact the SMC effectiveness. In the ACCESS-SMC study, it was estimated that 25.1 million doses of SMC were distributed monthly [26]. With the massive use of SMC, *pfdhps*540E prevalence must be carefully monitored in the Sahel region. The *pfdhps*540E level that could impact SMC effectiveness requires further investigation, for which the spatiotemporal maps developed in this study can be leveraged.

### Implications for IPTp-SP

WHO recommend IPTp-SP for all pregnant women living in areas of moderate-to-high malaria transmission in Africa, including areas with high-level SP resistance, determined by the prevalence of the quintuple mutant haplotype [15]. A recent comprehensive meta-analysis demonstrated that the protective effect of IPTp-SP against low birthweight was compromised in areas where *pfdhps*581G prevalence exceeded 10% (pooled mean prevalence of 37%) [22]. Our results suggest that the areas exceeding 50% *pfdhps*540E prevalence has expanded from a few isolated foci in 2000 to encompass most of East and South East Africa in 2020 (Fig 5), which may be due to an increased use of IPTp-SP [34]. This could constitute a growing ground for the sexutple mutation. The spatiotemporal map predictions indicate that *pfdhps*581G prevalence increased from less than 5% in most of Africa in 2000 to three main foci exceeding 37% in 2010: 1) Rwanda/East DRC/South West Uganda/North West Tanzania, 2) Nigeria and 3) North East Tanzania. In addition *pfdhps*581G prevalence exceeding 10% was observed in the region of Sudan/Eritrea/Ethiopia (Fig 5k). The spatioteporal maps indicate that *pfdhps*581G prevalence did not increase in the most recent predictions for 2020 (Fig 5j), which was supported by the analysis of the observed data where most sites, but not all, showed a significant decrease or no significant change between 2010 and 2020 (Figs 6 and 7). To be noted, recent and longitudinal *pfdhps*581G prevalence data were missing in many regions and especially the areas of higher prevalence which have higher uncertainty (Figs 4 and 7). These are cautiously optimistic findings for IPTp-SP, especially as IPTp-SP use has increased during this period. However, to confirm these predictions, more data are needed, specifically from the four foci with *pfdhps*581G prevalence exceeding 10% and high uncertainty (Figs 4f and 5k–5l). Moreover, effective and safe IPTp alternatives need to be identified and made available, starting in the parts of Africa where the prevalence of *pfdhps*581G exceeds 10% (Fig 5k–5i).

### Implications for IPTi-SP

While IPTp-SP has been shown to be effective even in some areas where *pfdhps*540E prevalence exceeds 90% [22], IPTi is recommended only in countries where *pfdhps*540E prevalence is lower than 50%. This recommendation came from the observation that IPTi-SP remained effective in areas with high prevalence of *pfdhfr* triple mutation and *pfdhps*437G, but the protection declined with an increased prevalence of the quintuple mutation and *pfdhps*540E. In Tanzania where the prevalence of quintuple mutations exceeded 90%, IPTi did not have a protective effect [37]. Another consideration is that infants are non-immune to malaria while pregnant women are semi-immune and could be able to clear residual parasites that are resistant to the IPT treatment. Thus a stricter threshold is needed for IPTi than IPTp, to ensure effectiveness. The spatiotemoral analyses demonstrated that the predicted *pfdhps*540E prevalence exceeding 50% was limited to a few foci in East and South East Africa in 2000, and has now, in 2020, spread to most of East and South East Africa (Fig 5). Regions with low-moderate prevalence of *pfdhps*540E (5–50%) expanded to include most of Central Africa, and low prevalence or complete absence of *pfdhps*540E mutations was restricted to the Sahel region in 2020. This development prevents effective IPTi-SP treatment in most East and South East Africa regions. Sierra Leone is so far the only country that has implememented IPTi at a large scale. IPTi-SP was included in the National Malaria Strategic Plan and implemented in all districts in 2018 with a 67.4% and 36.4% coverage of the first and third IPTi-SP dose, respectively [35].The spatiotemoral analysis in 2020 reveal one focus of low *pfdhps*540E prevalence (exceeding 5%) in West Africa, specifically in Sierra Leone and Liberia (Fig 5). This observation highlights the need to monitor *pfdhps*540E prevalence in districts that have implemented IPTi-SP to ensure continued effectiveness.

### Foci of resistance in East Africa

IPTp, IPTi and SMC are considered additional malaria control measures, complementing the core malaria control measures which are vector control with indoors residual spraying and/or insecticide treated nets, and diagnosis and treatment of confirmed cases with an ACT. In Africa, there was a recent emergence of K13 mutations associated with delayed parasite clearance, which could mediate artemisinin resistance (4–6). The emergence of K13 mutations spatially coincide with two of the predicted foci of *pfdhps*581G mutations in Rwanda/Uganda/DRC/Tanzania and Sudan/Eritrea/Somalia (Fig 5). Having parasites in the same area resistant to two cornerstones in malaria control, ACT and IPTp-SP, is of great concern. Studies need to be undertaken to further monitor the distribution of these mutations and to address if K13 mutations and *pfdhps*581G are carried by the same parasites.

### Near real-time data availability

When analyzing the time between sample collection and publication, we found that there was a 3-year median lag time. A reduction in this lag, could help to reduce uncertainty and increase the utility of the molecular data for informing drug policy decisions. In the WWARN SP Molecular Surveyor tool described here, prevalence data on *pfdhps* and *pfdhfr* markers were entered in a database and visualized [12]. The Surveyor credits all data sources and should encourage rapid sharing of information, perhaps even before full data are published. Similarly, other Surveyor tools display mutants in the propeller region of the K13 gene associated with slow parasite clearance after artemisinin treatment [44] and markers associated with resistance to ACT partner drugs [45]. These maps are regularly updated.

These kinds of initiatives can contribute to sharing and preparation of regional reports by researchers and national/regional surveillance programmes. Recently, journal editors and funders have specifically encouraged and even required sharing and use of data of public health importance pre-publication whilst maintaining credit and recognition for those who collect the data,. Using pre-prints or open review publishing platform could also help in this process. These initiatives have been propelled by urgent data needs in the COVID-19 pandemic [46–49].

### Data and uncertainty

Here we present marker prevalence data from 201 studies, conducted over 31 years, from heterogeneous data sources. To minimize heterogeneity, the data for the WWARN SP Molecular Surveyor were collated from the literature in a standardized approach with regards to the systematic literature search, inclusion of studies, data extraction, and presentation. Although data are collected extensively and regularly in some geographical sites, a main limitation of this study is the sparsity of data for most areas of the African continent. Previous work has been undertaken within the limitations of the data sparsity to analyse spatiotemporal trends in the *pfdhps* marker data [50], but did not attempt to make predictions where there was no data. The spatiotemporal model presented in this paper can predict the marker prevalence for locations that do not have data, using a method of approximation based on neighboring data points in time and/or space. However, the strength of the approximation decreases in regions that are increasingly further from sampling sites. An advantage of our model is that it was developed using a Bayesian framework which gives a natural measure for the uncertainty of the approximations. Uncertainty is higher in spatial regions where the prevalence of a marker is intermediate and not close to fixation (Figs 2c–2f and 3c–3g) and where prevalence is changing over time (Fig 5). The most recent maps also have higher uncertainty, unless the level of resistance is very high and has remained constant over time (i.e., fixed).

### Modelling to support surveillance

The current study demonstrates that the uncertainty of the median estimates of prevalence was higher when the marker prevalence was at an intermediate level. This is precisely the time when resistant parasites may be spreading and the need of malaria programmes and policy makers for accurate, current information is highest. Under these circumstances, a number of factors can reduce uncertainty and maximize the utility of spatiotemporal models. In particular, larger sample sets and higher geographic density of data points can lower the uncertainty of the estimations. In the current work, the relatively low number of samples that tested the prevalence of isolates that carried *pfdhps*581G is one source of uncertainty. Since resources in many malaria-endemic countries are limited, only focusing on filling data gaps may not be a useful approach. The level of uncertainty can be used for guiding surveillance to determine the locations for subsequent sample collection, referred to by Grist and colleagues as “smart surveillance” [51]. Thus, the map for a particular area can be strategically improved over time by utilizing both prevalence and uncertainty to identify sites where increased surveillance can be most informative: namely in the areas where uncertainty is high *and* where prevalence is intermediate or changing.

As big data algorithms and modelling predictions will play an increasingly important role in informing public health decision making, there is an urgent need to address how far extrapolation between sites is acceptable in terms of geographical distances and time. While prevalence of resistance markers can differ substantially in specific sites that are spatially and temporally close [52], public health decisions must usually be made at a national level. Further co-variates need to be explored and combined to fully understand the complex dynamics of the spread of resistance, for example human movement and behavior, density of human populations, drug pressure, environmental variables, and asymptomatic infections.

This study underlines the utility of sharing and combining molecular markers data and employing predictive modelling to highlight areas of concern that extend beyond national borders. We have built the WWARN SP Molecular Surveyor database to provide users with a standardized, current source of information on resistance marker distribution, a model that can be expanded to all validated markers associated with antimalarial resistance. With a possible emergence of artemisinin resistance in Rwanda, Uganda, Eritrea and Ghana, this approach could be easily expanded to better understand the evolution of this new threat.

Whenever appropriate data sets are available, a similar set of continuous spatiotemporal surface maps can be developed using this methodology. Application to other diseases could facilitate decisions in public health and guide future research in a particular region, country or Subregion. Timely sharing of molecular data is one key element in the utility of the approach.

## Supporting information

S1 Text SP Molecular Surveyor Data extraction and entry SOP. (DOCX)

S2 Text Supplementary methodology. (DOCX)

S1 Data Publications from Drug Resistance Maps database and SP Molecular Surveyor database. (XLSX)

S2 Data *pfdhps*581G prevalence Fisher’s exact test comparison between years in Fig 7. (XLSX)

S1 Fig Conditional dependency schematic for the geostatistical model applied to each of the three markers. Here, solid arrows represent conditional dependencies, the dashed arrow represents a deterministic relationship, the squares represent data and the circles/ellipses represent random variables.
(TIF)

S2 Fig Validation results for *pfdhps540*, showing (a) scatterplot of the predicted median prevalence from the validation models and observed prevalence and (b) probability-probability plot of the fraction of observations that fell within a predictive credible interval of a given size. The dashed red lines show a 1:1 reference line. In (a), the size of the dot is proportional to the sample size of the study.
(TIF)

S3 Fig Validation results for *pfdhps437*, showing (a) scatterplot of the predicted median prevalence from the validation models and observed prevalence and (b) probability-probability plot of the fraction of observations that fell within a predictive credible interval of a given size. The dashed red lines show a 1:1 reference line. In (a), the size of the dot is proportional to the sample size of the study.
(TIF)

S4 Fig Validation results for *pfdhps581*, showing (a) scatterplot of the predicted median prevalence from the validation models and observed prevalence and (b) probability-probability plot of the fraction of observations that fell within a predictive credible interval of a given size. The dashed red lines show a 1:1 reference line. In (a), the size of the dot is proportional to the sample size of the study.
(TIF)

S1 Video *pfdhps*437G data collection over time. The video shows the time course of data collection for *pfdhps*437G over the period of 1990 to 2020. Data visualized in each year shows studies conducted before or during the year associated with the map. National shapefiles were obtained from the Malaria Atlas Project (MAP; https://malariaatlas.org/) under their open access policy (https://malariaatlas.org/open-access-policy/) and no changes were made.
(MP4)

S2 Video *pfdhps*540E data collection over time. The video shows the time course of data collection for *pfdhps*540E over the period of 1990 to 2020. Data visualized in each year shows studies conducted before or during the year associated with the map. National shapefiles were obtained from the Malaria Atlas Project (MAP; https://malariaatlas.org/) under their open access policy (https://malariaatlas.org/open-access-policy/) and no changes were made.
(MP4)

S3 Video *pfdhps*581G data collection over time. The video shows the time course of data collection for *pfdhp*s581G over the period of 1990 to 2020. Data visualized in each year shows studies conducted before or during the year associated with the map. National shapefiles were obtained from the Malaria Atlas Project (MAP; https://malariaatlas.org/) under their open access policy (https://malariaatlas.org/open-access-policy/) and no changes were made.
(MP4)

S4 Video Spatiotemporal modelling of *pfdhps*437G mutation prevalence. The video shows the median of the posterior predictive distribution for *pfdhps*437G mutation prevalence over 1990 to 2020. Data visualized in each year shows studies conducted before or during the year associated with the map, the size of the dots is proportional to the study sample size and the colour is representative of the observed marker prevalence. National shapefiles were obtained from the Malaria Atlas Project (MAP; https://malariaatlas.org/) under their open access policy (https://malariaatlas.org/open-access-policy/) and no changes were made.
(MP4)

S5 Video Spatiotemporal modelling of *pfdhps*540E mutation prevalence. The video shows the median of the posterior predictive distribution for *pfdhps*540E mutation prevalence over 1990 to 2020. Data visualized in each year shows studies conducted before or during the year associated with the map, the size of the dots is proportional to the study sample size and the colour is representative of the observed marker prevalence. National shapefiles were obtained from the Malaria Atlas Project (MAP; https://malariaatlas.org/) under their open access policy (https://malariaatlas.org/open-access-policy/) and no changes were made.
(MP4)

S6 Video Spatiotemporal modelling of *pfdhps*581G mutation prevalence. The video shows the median of the posterior predictive distribution for *pfdhps*581G mutation prevalence over 1990 to 2020. Data visualized in each year show studies conducted before or during the year associated with the map, the size of the dots is proportional to the study sample size and the colour is representative of the observed marker prevalence. National shapefiles were obtained from the Malaria Atlas Project (MAP; https://malariaatlas.org/) under their open access policy (https://malariaatlas.org/open-access-policy/) and no changes were made.
(MP4)
