# Supplementary material for: Spatiotemporal spread of Plasmodium falciparum mutations for resistance to sulfadoxine-pyrimethamine across Africa, 1990–2020
Source: PLoS Comput Biol. 2022 Aug 11;18(8):e1010317. doi: 10.1371/journal.pcbi.1010317 (PMC9371298; doi:10.1371/journal.pcbi.1010317)
Supplement: S1 Text — (DOCX) [file pcbi.1010317.s001.docx]

**Data extraction and entry for the**

**ACT Partner Drug Molecular Surveyor**

**And**

**SP Molecular Surveyor**

**SOP version 1.3**

The following document was created to guide extraction marker prevalence data from publications and data entry in the form for molecular data. The document includes inclusion and exclusion criteria for publications, a list of markers displayed in the Molecular Surveyor, decisions on data entry and display and a step-by-step guide on how to enter data in the Molecular form.

Author: Sabina Otienoburu

Version: 1.3

Date: January 2020

Versions

1.1 Adding plasmepsin copy number

1.2 Reinforcing to use Google Maps for lat and lon and the batch geocoder Doogal.

1.3 Removing geocoder Doogal, which does no longer work internationally, exchange with instructions for Google maps.

**Inclusion criteria for publications:**

- At least one *pfdhfr* or *pfdhps* genotype/haplotype from isolate/infection
- Original data
- Baseline/pre-treatment infections
- Marker prevalence must be linked to study site/country

**Exclusion criteria for publications:**

- Publication not accessible
- Publication in another language than English, French, Spanish or Italian
- Review articles
- Prevalence data that have been previously published (data from original publication is included)
- Cultured strains (e.g. 3D7), genetically manipulated strains, strains that have been adapted to long-term culture in vitro (not representative of original infection)
- Non-human *P. falciparum* infections (Anopheles, monkeys)
- Post-treatment infections
- Biased selection of baseline samples (e.g. only treatment failures, half resistant and half sensitive in vitro tested isolates)
- Regionally pooled marker prevalence data (i.e. from West Africa) where the origin of the infection cannot be deduced on at least country level
- Marker prevalence (%) presented in article, without sample size
- parasites isolated from placenta
- patients with severe malaria who received treatment previously
- results previously published using another method
- not possible to distinguish which isolates originates from treated and untreated patients
- case study of traveler that had self-medicated

**Example of studies which would be excluded:**

- - patients with severe malaria who received treatment previously
  - results previously published using another method
  - data only presented as haplotypes of *pfcrt* AND *pfmdr1* grouped together, not possible to separate marker prevalence from single genes
  - case study of traveler that had self-medicated
  - pooled analysis of previously published data
  - prevalence that includes both pre- and posttreatment isolates

**Molecular Surveyor Data Extraction and Entry**

In summary the data extraction contains the following steps that are detailed below:

1. Make the pub med search ‘malaria AND (pfcrt OR pfmdr1 OR plasmepsin2 OR dhfr OR dhps OR pfdhfr OR pfdhps OR “molecular marker” OR “molecular markers”)‘ to identify publications
2. Examine publications and include accessible, original publications of baseline/pre-treatment samples with marker prevalence data, including sample size, linked to study site/country. Study year is desirable but not essential.
3. Use the Molecular Surveyor data entry form to enter marker prevalence data *per site per year*. Verify how mixed infections are presented in the publication: included/excluded in the prevalence or presented separately. Enter the prevalence of the wild-type, mutant and mixed infections separately for ACT Partner Drug markers. For SP markers, enter the prevalence of mutant and mixed infections separately.

**Publication entry**

Enter the PubMedID and fields for author, title, publication year and journal will automatically populate.

Enter the PubMed URL.

**Study site entry**

*Create an entry per site per year (e.g. data from two sites for years 2010, 2011, 2012 = six entries)*

**StudyID = pubmed ID** automatically populated

**Rn = entry number counter** automatically populated

**Start year, end year** Year that data collection started and ended. If the study only spanned one year, enter the same year in both start and end.

**Estimated location = yes** Study site is not specified, for example for travelers where you only know the country of infection or multisite studies where the data is not presented by site.

**Estimated year = yes** Year of data collection is not specified in the publication. Estimated to “publication year – 3 years”

**Estimated prevalence = yes** Prevalence data is extracted/calculated from a graph where an approximate prevalence can be read. Only if sample size is known

**Location**

Select existing location or create a new site with coordinates

Coordinates can be found at: [www.google.com/maps](http://www.google.com/maps)

Search on Study site, right click on location and click on ‘What is here’ to get coordinates

**Notes** Enter comments regarding the study of interest for the user, such as if mixed infections were excluded.

**Marker prevalence entry**

*Marker prevalence data is entered per site per year.* *Enter the prevalence of the wild-type (e.g. pfcrt K76), mutant (e.g. pfcrt 76T) and mixed infections (e.g. pfcrt 76K/T) separately in the form. Calculate missing marker prevalences if data are not presented in the publication, if possible. Extract data from all the markers in the list below.*

Select marker (gene, position, amino acid)

Enter number of tested and present samples of the genotype

- 1. Mixed infections
     1. If mixed infection prevalence is presented separately or if you can calculate mixed infection prevalence from the numbers in the publication, enter mixed infections separately.
     2. If prevalence of mixed infections was included in the prevalence of one marker and you are not able to separate it, select ‘MixedIncl’.
  2. Haplotypes
     1. If only haplotypes are presented in the article, calculate the prevalence of single markers, i.e. *pfcrt* K76 and 76T and *pfmdr1* N86 and 86Y and enter them, if possible.

**Decisions and guidance on data entry and display**

**Data extraction**

- Data in the publication is extracted “as is”, referred publications are not explored to find missing information; study year, study site etc.

**Study year**

- If prevalence data are available per year, enter the information per year. If it is available over a range of years, enter a range.
- If there is no information in the publication of when the study was performed, set the year to “publication year – 3 years” and check the box ‘Estimated year’.

**Study site**

- In a multisite study, add multiple sites and enter prevalence data by site.
- If there are multiple study sites and the marker prevalence cannot be separated by site, the combined prevalence for all study sites is entered for one of the study sites. Select ‘Estimated location’, ‘Create a new location’ and enter the name of all study sites. Enter the coordinates for one of the study sites. The marker prevalence will be displayed with a pin pointing at the study site for which you entered the coordinates, however all study sites will be displayed in the box.
- If there is no information on study site, but just country of origin, for example in travelers, select the capital of the country as location. Select ‘Estimated location’. The marker prevalence is displayed with a pin pointing at the capital of the country.

**Sample size**

- Sample size must be known for inclusion of data in the Molecular Surveyor.
- Add marker prevalence per site per year if sample size is available per site per year.
- If sample size is only available for a range of years, enter data over a range of years, even though specific marker prevalence data may be available per year.
- If sample size is only available as a combined sample size for several study sites, enter the combined prevalence of all sites for one of the sites in the study, even though specific marker prevalence data may be available per site. Select ‘Estimated location’.

**Marker prevalence**

- Always enter the prevalence of the wild-type (e.g. *pfcrt* K76), mutant (e.g. *pfcrt* 76T) and mixed infections (e.g. *pfcrt* 76K/T) separately when possible.
- If the prevalence of only one allele (e.g. *pfcrt* 76T) is presented in the publication, the prevalence of the other allele (*pfcrt* K76) is calculated from the presented prevalence, if the mixed infections (*pfcrt* 76K/T) are accounted for (prevalence of mixed infections presented/mixed infections excluded/mixed infection included in the presented prevalence/no mixed infections identified).
- If mixed infections were included in the prevalence of one marker and you are not able to enter them separately, enter the combined prevalence of marker+ mixed infections and select ‘MixedIncl’.
- If only prevalence of haplotypes (e.g. *pfcrt* 72-76 or *pfmdr1* 86 184 1246) is presented in the publication, the prevalence of the single loci (*pfcrt* 76 or *pfmdr1* 86) is calculated from the haplotype prevalence, if there is nothing in the publication indicating that the prevalence of the haplotype is not representative for the prevalence of the single loci.
- If there was no information about mixed infections in the publication and the total prevalence of wild-type+mutant allele was > 100%, it was assumed that mixed infections were included in the prevalence of both the wild-type and the mutant allele.
- If the number of samples positive for a marker is not available in the article, but only the prevalence (%), in text or in a graph, and sample size is available, enter the calculated number samples positive for the marker.
- If the marker prevalence is presented in several groups, for example per treatment arm, calculate the total marker prevalence for both arms (per site and per year) and enter the total number of tested and present samples

**List of markers included in the Molecular Surveyor**

ACT partner drug markers

*pfcrt* K76

*pfcrt* 76T

*pfcrt* 76K/T

*pfcrt* 72,74-76 CXXK

*pfcrt* 72,74-76 CXXT

*pfcrt* 72,74-76 SXXT

*pfmdr1* N86

*pfmdr1* 86Y

*pfmdr1* 86N/Y

*pfmdr1* Y184

*pfmdr1* 184F

*pfmdr1* 184Y/F

*pfmdr1* D1246

*pfmdr1* 1246Y

*pfmdr1* 1246D/Y

*pfmdr1* NFD

*pfmdr1* YYY

*pfmdr1* mixes for any of the above combination of three SNPs (86+184+1246)

*pfmdr1* NXXXD

*pfmdr1* YYXXY

*pfmdr1* mixes for any of the above combinations of the five SNPs (86+184+1034+1042+1246)

*pfmdr1* copy number = 1

*pfmdr1* copy number >1

*plasmepsin2* copy number = 1

*plasmepsin2* copy number > 1

SP markers

*dhps*+dhps 51I-59R-108N-437G-540E

*dhps*+dhps 51I-59R-108N-437G-540E-581G

*dhps*+dhps 51I-59R-108N-437G-540E-A581

*dhps* 51I-59R-108N

*dhps* 59I-C59-108N

*dhps* N51-59R-108N

*dhps* 50R

*dhps* 51I

*dhps* 59R

*dhps* 108N

*dhps* 108T

*dhps* 164L

*dhps* 437G-540E-581G

*dhps* 437G-540E

*dhps* 437G-540E-A581

*dhps* 436A

*dhps* 437G

*dhps* 540E

*dhps* 581G

*dhps* 613S

*dhps* 613T
