# Supplementary material for: Spatiotemporal spread of Plasmodium falciparum mutations for resistance to sulfadoxine-pyrimethamine across Africa, 1990–2020
Source: PLoS Comput Biol. 2022 Aug 11;18(8):e1010317. doi: 10.1371/journal.pcbi.1010317 (PMC9371298; doi:10.1371/journal.pcbi.1010317)
Supplement: S2 Text — (DOCX) [file pcbi.1010317.s002.docx]

**S2 Text**

1. Data extraction

Data on *pfdhfr* and *pfdhps* genotype and haplotype prevalences (number of samples tested and number of mutant samples identified), PubMedID and publication information, year and geo-position (country, site, latitude and longitude) were extracted from publications. The number of samples with pure mutant alleles and mixed mutant and wild-type alleles were entered separately in the database. Markers associated with resistance to SP treatment were selected to for extraction: *pfdhfr* 51, 59, 108, 164, and *pfdhps* 437, 540, 581, 613. If latitude/longitude were not reported in the article, geocoding was performed using online tools [www.gpsvisualizer.com/geocoder/ or [www.google.com/maps](http://www.google.com/maps)]. Data were entered in an online form and converted to relational tables in a SQL database. The main WWARN SQL database is a fault-tolerant database, which replicates data in different data centres to achieve high availability and durability. Daily back-ups are also applied in order to minimise data loss.

The following rules guide the process of data extraction and entry:

- Data are extracted and entered per site per year, if this information is available.
- Sample size must be specified per site and marker.
- Articles are scanned particularly to identify reports of polyclonal infections. If polyclonal infections are present in the data set, they can be entered separately, or included in either the mutant and wild type categories or both, depending on the information given in the publication.
- If no sample collection date is reported, the year of sample collection is estimated to 3 years prior to the publication year.
- If the marker prevalence is only presented in a graph in the article, the prevalence for each genotype is estimated based on the graph.
- If only the prevalence of haplotypes (e.g. *pfdhps 437-540*) is presented in the publication, the prevalence of the single locus (*pfdhps* 540) is calculated from the haplotype prevalence, if all identified haplotypes were accounted for in the publication.

1. Geostatistical Model

The mathematical model was developed using a model-based geostatistics (MBG) framework (1) and parameters were estimated in a Bayesian inference setting using Markov chain Monte Carlo (MCMC) simulation (2). A similar approach was previously used to predict *pfdhps540E* levels across Africa from 1990 to 2010 (3). The Bayesian MBG approach allows for spatial prediction within the generalised linear modelling framework as well as for parameter estimation and quantification of uncertainty.

2.1 Probability model for *pfdhps*437G, *pfdhps*540E and *pfdhps*581G markers

A schematic representation of the statistical model used in this work is given in S1 Fig, which shows the conditional dependencies in the geostatistical model applied to each of the three markers. The number of individuals in the $i^{th}$study, conducted at location $\boldsymbol{x}_{i}$ in year $t_{i}$, that were positive for the marker ($N_{i}^{+}$) was taken to be binomially distributed, given the number of individuals tested in the $i^{th}$study ($N_{i}$) and the probability $p(\boldsymbol{x}_{i},t_{i})$:

$N_{i}^{+} \sim Binomial\left( N_{i}, p(\boldsymbol{x}_{i},t_{i}) \right)$.

The probability, $p(\boldsymbol{x},t)$, at location$\boldsymbol{x}$and time $t$, was modeled as the inverse logit transformation of the sum of a random field,$Z(\boldsymbol{x},t)$, and an unstructured random component, $\epsilon(\boldsymbol{x},t)$:

$p\left( \boldsymbol{x},t \right)=\mathrm{logit}^{-1}\left( f\left( \boldsymbol{x},t \right) \right)= \mathrm{logit}^{-1}\left( Z\left( \boldsymbol{x},t \right)+\epsilon\left( \boldsymbol{x},t \right) \right)$.

The unstructured components,$\epsilon(\boldsymbol{x},t)$, were assumed to be independent and identically Normally distributed variables with zero mean and variance$V$. The random field, $Z(\boldsymbol{x},t)$, was modelled as a Gaussian process, with mean function$\mu(\boldsymbol{x},t)$ and covariance function $C(\boldsymbol{x},t)$:

$Z\left( \boldsymbol{x},t \right)|\boldsymbol{\theta}_{M},\boldsymbol{\theta}_{C} \sim GP(\mu\left( \boldsymbol{x},t \right), C(\boldsymbol{x},t))$,

where $\boldsymbol{\theta}_{M}$ and $\boldsymbol{\theta}_{C}$ are vectors of parameters that specify the mean and covariance functions, respectively.

In this work, it was assumed that the mean function varies linearly in time and with malaria transmission intensity:

$\mu\left( \boldsymbol{x},t \right)= \beta_{0}+ \beta_{1}t+ \beta_{2}m(\boldsymbol{x}, t\boldsymbol{)}$,

where $\boldsymbol{\theta}_{M}=\left\{ \beta_{0},\beta_{1},\beta_{2} \right\}$, $t$ is time measured since 1990 and $m(\boldsymbol{x}, t)$ is *P. falciparum* transmission intensity from 2010-2017, as estimated by the spatiotemporal models developed by the Malaria Atlas Project (MAP) (4). Note that for years before 2010, we use the 2010 transmission intensity and for years after 2017, we use the 2017 transmission intensity.

The covariance function was chosen to be a version of the spatio-temporal structure advocated by Stein (5) and adopted previously by Hay *et al.* (6), Gething *et al.* (7) and Flegg *et al.* (8). The covariance between two studies conducted at locations $\boldsymbol{x}_{i}$ and $\boldsymbol{x}_{j}$ and in years $t_{i}$ and $t_{j}$ was taken to be:

$C\left( \boldsymbol{x}_{\boldsymbol{i}},t_{i}, \boldsymbol{x}_{\boldsymbol{j}},t_{j} \right)= \sigma^{2}\gamma\left( 0 \right)\frac{{\Delta x}^{\gamma(\Delta t)}\kappa_{\gamma(\Delta t)}(\Delta x)}{2^{\gamma\left( \Delta t \right)-1}\Gamma(\gamma\left( \Delta t \right)+1)}$,

where $\Gamma$ is the gamma function,$\kappa_{\gamma}$ is the modified Bessel function of the second kind of order$\gamma$, $\Delta t=\left| t_{i}-t_{j} \right|$ and $\gamma\left( \Delta t \right)=\left( 2\rho+2\left( 1-\rho\right)^{\frac{-\Delta t}{\phi_{t}}} \right)^{-1}.$The distance$\Delta x$ is given by:

$\Delta x= \frac{2\sqrt{\gamma\left( \Delta t \right)}D_{GC}\left( \boldsymbol{x}_{i}, \boldsymbol{x}_{j} \right)}{\phi_{x}}$,

where $D_{GC}\left( \boldsymbol{x}_{i}, \boldsymbol{x}_{j} \right)$is the great circle distance between locations $\boldsymbol{x}_{i}$ and $\boldsymbol{x}_{j}$. In the notation adopted here, the parameter $\phi_{t}$refers to the temporal scale factor, $\rho$to the temporal limiting correlation, $\sigma^{2}$to the partial sill and $\phi_{x}$to the spatial range. The covariance parameters were thus ${\boldsymbol{\theta}_{C}=\{\phi}_{t}, \phi_{x}, \rho, \sigma^{2}\}.$ $V$ is the nugget variance.

The joint probability model for the $n$marker observations, the field components and the model parameters was therefore given by:

$p(\boldsymbol{N}^{\boldsymbol{+}}, f, \boldsymbol{\theta}_{M}, \boldsymbol{\theta}_{C}, V)= \prod_{i=1}^{i=n} p\left( N_{i}^{+} | f\left( \boldsymbol{x}_{i}, t_{i} \right), N_{i} \right)p\left( f | \boldsymbol{\theta}_{M}, \boldsymbol{\theta}_{C}, V \right)p\left( \boldsymbol{\theta}_{M} \right)p\left( \boldsymbol{\theta}_{C} \right)p(V)$.

2.2 Prior specification

Priors were specified for the mean and covariance parameters $\left\{ \boldsymbol{\theta}_{M}, \boldsymbol{\theta}_{C}, V \right\}=\{\beta_{0},\beta_{1},\beta_{2},\phi_{t}, \rho, \sigma^{2}, \phi_{x}, V\}$ . The logarithm of the partial sill ($\sigma^{2}$) and the spatial range ($\phi_{x}$) were assigned skew-normal priors, while the temporal scale ($\phi_{t}$) was given a relatively vague Exponential prior. The temporal limiting correlation ($\rho$) was assigned a uniform prior on $\left[ 0,1 \right]$ while noninformative priors were specified for the mean coefficients:

$p\left( \beta_{0},\beta_{1},\beta_{2} \right)\propto1$.

Finally, the inverse of the nugget variance ($V$) was assigned a diffuse Gamma prior.

2.3 Implementation

The implementation of the model proceeds with two main steps: inference and prediction, as detailed below.

2.3.1 Parameter estimation (inference stage)

In the parameter estimation stage, the output of the model was the posterior probability distribution of the $(n+8)$model parameters ($\left\{ \boldsymbol{\theta}_{M}, \boldsymbol{\theta}_{C}, V, f\left( \boldsymbol{x}_{1}, t_{1} \right), f\left( \boldsymbol{x}_{2}, t_{2} \right), \ldots, f\left( \boldsymbol{x}_{n}, t_{n} \right) \right\}$), given the observed data. Samples were drawn from the posterior distribution of the model mean and covariance parameters ($\left\{ \boldsymbol{\theta}_{M}, \boldsymbol{\theta}_{C}, V \right\}$) and the random field ($f\left( \boldsymbol{x}_{i}, t_{i} \right)$) at each location where the marker data was available, using an MCMC approach. The MCMC algorithm was implemented in the Python package PyMC (9). PyMC is an open-source Python module that implements Bayesian statistical models and fitting algorithms, including MCMC.

2.3.2. Spatiotemporal mapping (prediction stage)

In the prediction stage, the output was the posterior predictive distribution of the prevalence of the marker at each space-time point of interest; here each location on a 5 x 5 km grid in *P. falciparum* spatial limits of Africa (defined by MAP) from 1990-2020. From the output of the inference stage, parameter values were available for the sample from the posterior $\left\{ \beta_{0}^{j},\beta_{1}^{j}, \beta_{2}^{j},\phi_{t}^{j}, \rho^{j},{\sigma^{2}}^{j}, \phi_{x}^{j}, V^{j} \right\}, j=1, \ldots, m$ and for $f^{j}\left( \boldsymbol{x}_{i}, t_{i} \right), j=1, \ldots, m$ for each of the data locations ($i=1, \ldots, n$).

To generate a predictive map for a year of interest, for each of the samples ($j=1, \ldots, m$), for each of the prediction locations on a 5 x 5 km grid, the conditional distribution of the random field, was sampled from a multivariable Normal distribution, conditional on $\left\{ \beta_{0}^{j},\beta_{1}^{j}, \beta_{2}^{j},\phi_{t}^{j}, \rho^{j},{\sigma^{2}}^{j}, \phi_{x}^{j}, V^{j} \right\}$ and the $f^{j}\left( \boldsymbol{x}_{i}, t_{i} \right), i=1, \ldots, n$. Repeating this for each of the $m$ samples formed the set of marker prevalence samples for this space-time location, for which the median and standard deviation was found. Repeating for each prediction location on a 5 x 5 km grid resulted in median and standard deviation maps of marker prevalence.

1. Validation procedures and results

Model validity was assessed by dividing the dataset (for each maker, performed separately) into 10 random subsets of data. For each subset, the model was rerun with the 10% of data withheld. The median predictions at the withheld points were then used as validation for the model. This was repeated for each of the 10 subsets of data and then for each of the markers. In the main manuscript (Table 2) we report the correlation coefficient as a measure of linear association between the median predicted values and the observed prevalences, the mean error as a measure of bias and the mean absolute error as a measure of average accuracy. Overall, there is good agreement between the observed and predicted prevalences (especially for *pfdhps540*). There is a small amount of bias and the accuracy is reasonable (especially for *pfdhps540*).

S2 Fig, S3 Fig and S4 Fig show the validation results for the *pfdhps540*, *pfdhps437* and *pfdhps581* markers, respectively. Again, we see good agreement between the observed and predicted prevalences (especially for *pfdhps540*) and the reliability of the credible intervals was strong.

**References**

1. Diggle PJ, Tawn JA, Moyeed RA. Model-based geostatistics. Journal of the Royal Statistical Society: Series C (Applied Statistics). 1998;47(3):299–350.

2. Gilks WR, Richardson S, Spiegelhalter D. Markov Chain Monte Carlo in Practice. CRC Press; 1995. 522 p.

3. Bhatt S, Weiss DJ, Cameron E, Bisanzio D, Mappin B, Dalrymple U, et al. The effect of malaria control on Plasmodium falciparum in Africa between 2000 and 2015. Nature. 2015 Oct 8;526(7572):207–11.

4. Weiss DJ, Lucas TCD, Nguyen M, Nandi AK, Bisanzio D, Battle KE, et al. Mapping the global prevalence, incidence, and mortality of Plasmodium falciparum, 2000–17: a spatial and temporal modelling study. The Lancet. 2019 Jul 27;394(10195):322–31.

5. Stein ML. Space–Time Covariance Functions. Journal of the American Statistical Association. 2005 Mar 1;100(469):310–21.

6. Hay SI, Guerra CA, Gething PW, Patil AP, Tatem AJ, Noor AM, et al. A world malaria map: Plasmodium falciparum endemicity in 2007. PLoS Med. 2009 Mar 24;6(3):e1000048.

7. Gething PW, Patil AP, Smith DL, Guerra CA, Elyazar IR, Johnston GL, et al. A new world malaria map: Plasmodium falciparum endemicity in 2010. Malar J. 2011 Dec 20;10:378.

8. Flegg JA, Patil AP, Venkatesan M, Roper C, Naidoo I, Hay SI, et al. Spatiotemporal mathematical modelling of mutations of the dhps gene in African Plasmodium falciparum. Malar J. 2013 Jul 17;12:249.

9. Patil A, Huard D, Fonnesbeck CJ. PyMC: Bayesian Stochastic Modelling in Python. J Stat Softw. 2010 Jul;35(4):1–81.
