# Supplementary figures and images for: Spatiotemporal spread of Plasmodium falciparum mutations for resistance to sulfadoxine-pyrimethamine across Africa, 1990–2020
Source: PLoS Comput Biol. 2022 Aug 11;18(8):e1010317. doi: 10.1371/journal.pcbi.1010317 (PMC9371298; doi:10.1371/journal.pcbi.1010317)

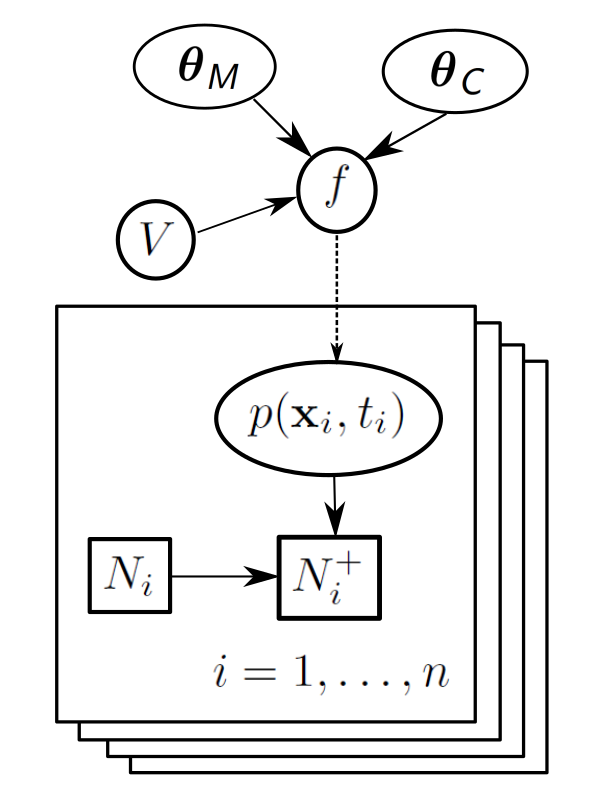

Supplement: S1 Fig — Here, solid arrows represent conditional dependencies, the dashed arrow represents a deterministic relationship, the squares represent data and the circles/ellipses represent random variables. (TIF) [file pcbi.1010317.s005.tif]

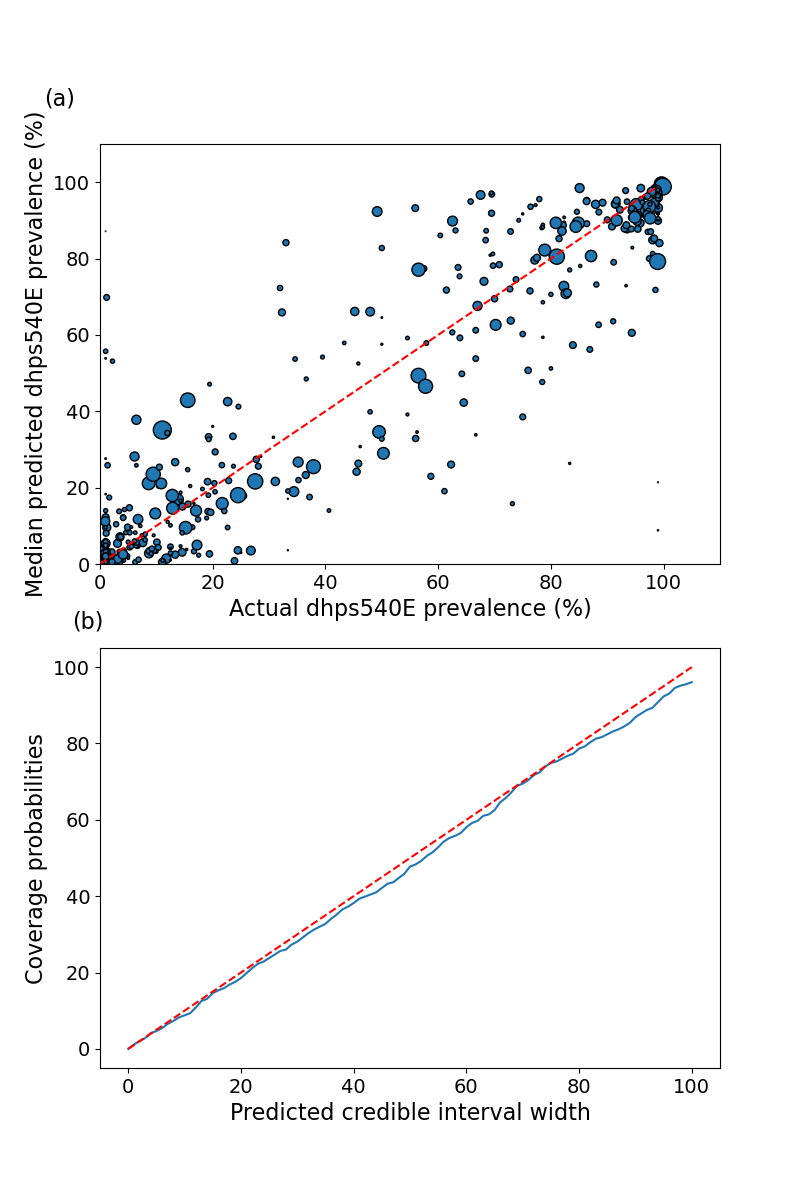

Supplement: S2 Fig — Validation results for pfdhps540, showing (a) scatterplot of the predicted median prevalence from the validation models and observed prevalence and (b) probability-probability plot of the fraction of observations that fell within a predictive credible interval of a given size. The dashed red lines show a 1:1 reference line. In (a), the size of the dot is proportional to the sample size of the study. (TIF) [file pcbi.1010317.s006.tif]

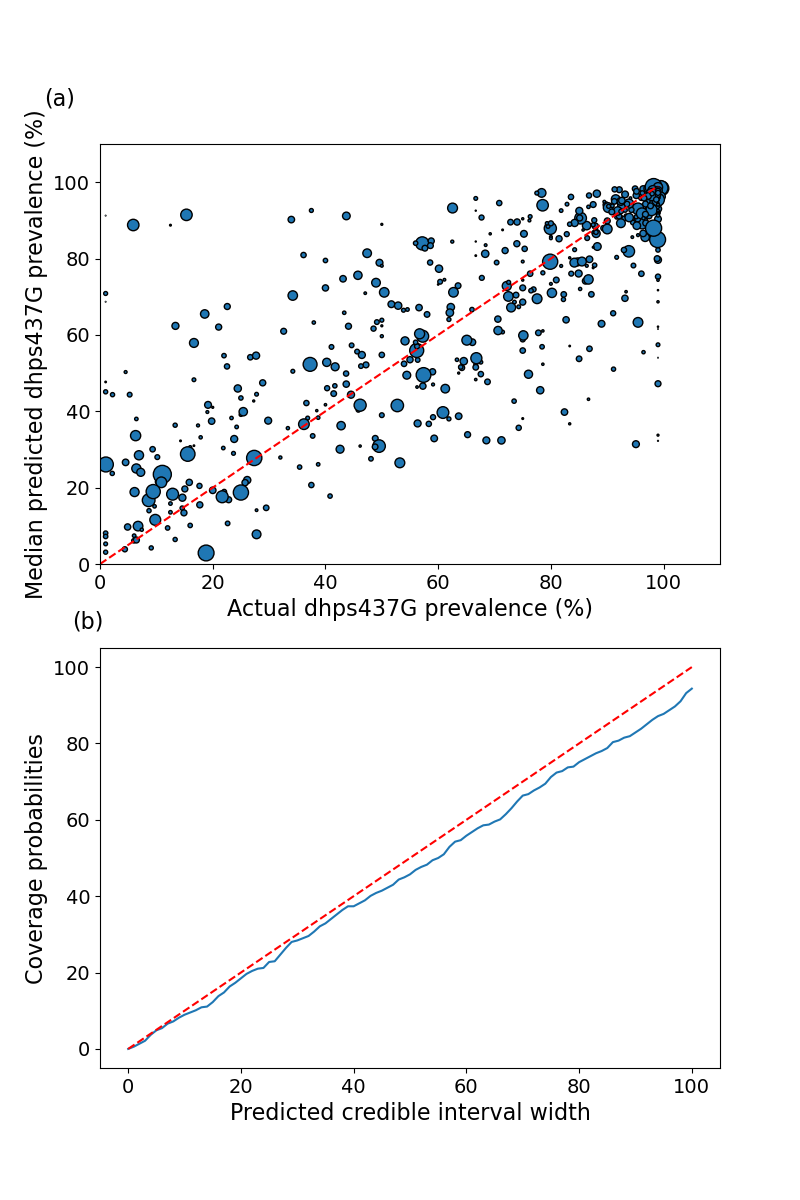

Supplement: S3 Fig — Validation results for pfdhps437, showing (a) scatterplot of the predicted median prevalence from the validation models and observed prevalence and (b) probability-probability plot of the fraction of observations that fell within a predictive credible interval of a given size. The dashed red lines show a 1:1 reference line. In (a), the size of the dot is proportional to the sample size of the study. (TIF) [file pcbi.1010317.s007.tif]

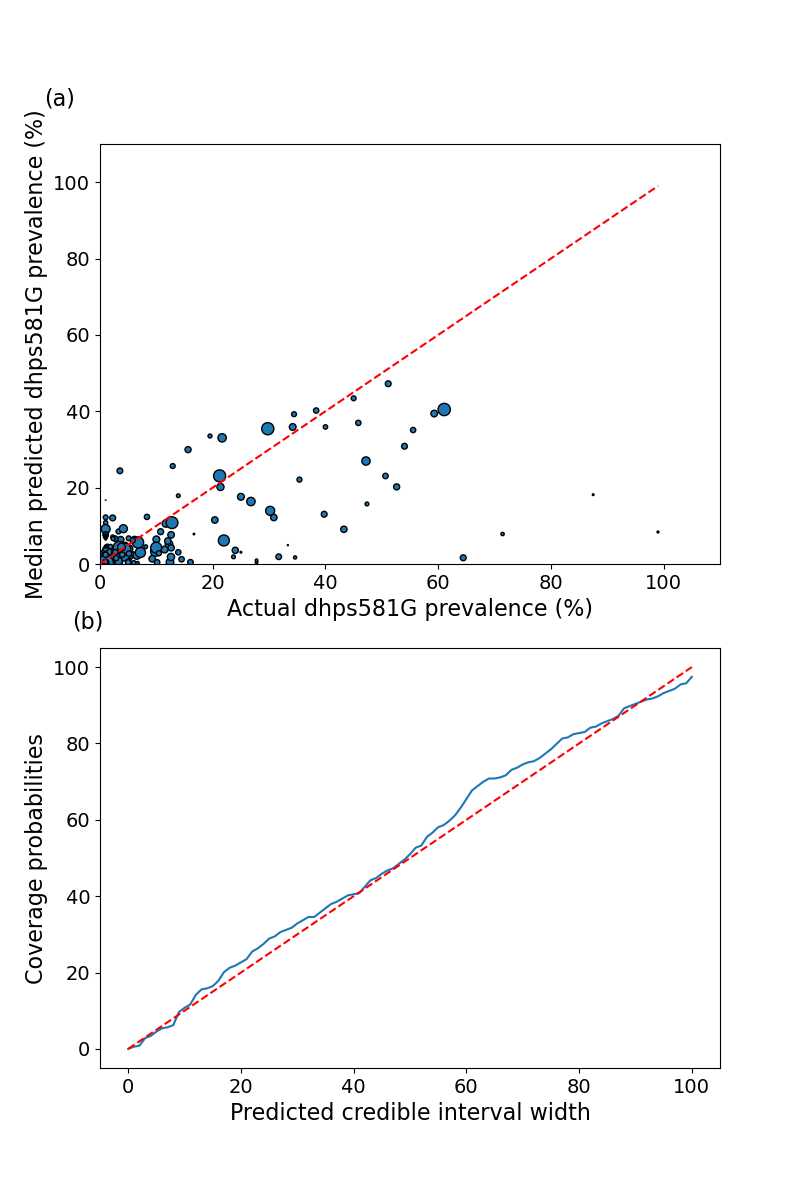

Supplement: S4 Fig — Validation results for pfdhps581, showing (a) scatterplot of the predicted median prevalence from the validation models and observed prevalence and (b) probability-probability plot of the fraction of observations that fell within a predictive credible interval of a given size. The dashed red lines show a 1:1 reference line. In (a), the size of the dot is proportional to the sample size of the study. (TIF) [file pcbi.1010317.s008.tif]
